# Targeted Protein Degradation (TPD) for Immunotherapy: Understanding Proteolysis Targeting Chimera-Driven Ubiquitin-Proteasome Interactions

**DOI:** 10.1021/acs.bioconjchem.4c00253

**Published:** 2024-07-11

**Authors:** Rajamanikkam Kamaraj, Subhrojyoti Ghosh, Souvadra Das, Shinjini Sen, Priyanka Kumar, Madhurima Majumdar, Renesa Dasgupta, Sampurna Mukherjee, Shrimanti Das, Indrilla Ghose, Petr Pavek, Muruga Poopathi Raja Karuppiah, Anil A. Chuturgoon, Krishnan Anand

**Affiliations:** † Department of Pharmacology and Toxicology, Faculty of Pharmacy, Charles University in Prague, Heyrovskeho 1203, 50005 Hradec Kralove, Czech Republic; ‡ Department of Chemical Pathology, School of Pathology, Faculty of Health Sciences, University of the Free State, Bloemfontein, Free State 9300, South Africa; § Department of Biotechnology, Indian Institute of Technology Madras, Chennai 600036, India; ∥ Department of Biotechnology, Heritage Institute of Technology, Kolkata 700107, India; ⊥ Department of Chemistry, School of Physical Sciences, Central University of Kerala, Tejaswini Hills, Periye, Kasaragod District, Kerala 671320, India; # Discipline of Medical Biochemistry, School of Laboratory Medicine and Medical Sciences, College of Health Sciences, Howard College Campus, University of KwaZulu-Natal, Durban 4041, South Africa

## Abstract

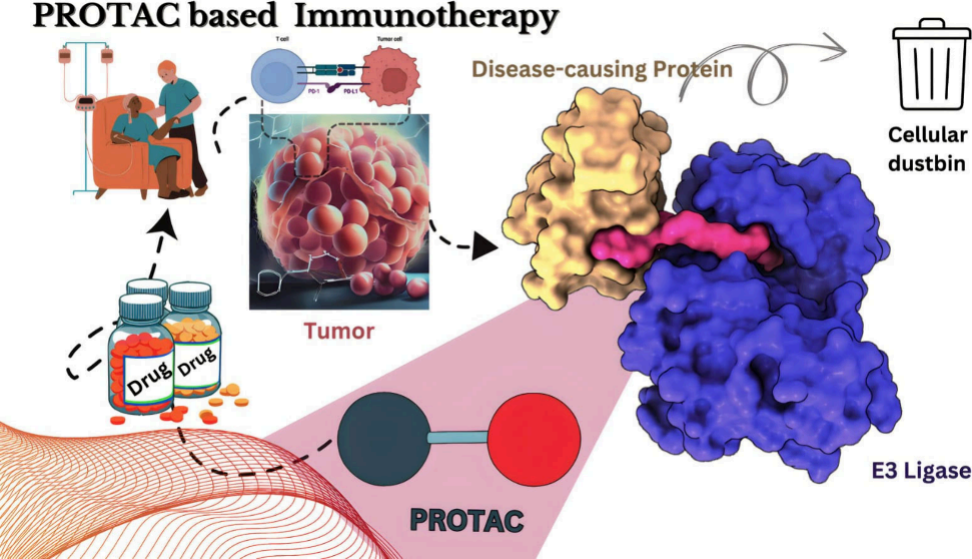

Targeted protein degradation or TPD, is rapidly emerging as a treatment that utilizes small molecules to degrade proteins that cause diseases. TPD allows for the selective removal of disease-causing proteins, including proteasome-mediated degradation, lysosome-mediated degradation, and autophagy-mediated degradation. This approach has shown great promise in preclinical studies and is now being translated to treat numerous diseases, including neurodegenerative diseases, infectious diseases, and cancer. This review discusses the latest advances in TPD and its potential as a new chemical modality for immunotherapy, with a special focus on the innovative applications and cutting-edge research of PROTACs (Proteolysis TArgeting Chimeras) and their efficient translation from scientific discovery to technological achievements. Our review also addresses the significant obstacles and potential prospects in this domain, while also offering insights into the future of TPD for immunotherapeutic applications.

## 1 Introduction

Immunotherapy, also known as biological therapy, is a type of therapeutic approach that involves manipulating the immune response to treat diseases. It encompasses methods aimed at either enhancing or suppressing immune responses. Activation immunotherapies are intended to trigger or strengthen the immunological network, whereas suppression immunotherapies are considered to diminish or inhibit the activity of the immune system. Currently, under intense scrutiny, immunotherapy holds significant promise as a potential avenue for treating various cancer types.^1,2^

A number of cancers have shown significant success with immunomodulation, a standard treatment that can be used alone or combined with radiotherapy and chemotherapy.^3^ T cells express coinhibitory receptors such as Programmed Cell Death 1 (PD-1) and Cytotoxic T Lymphocyte Antigen 4 (CTLA-4) on their surface to regulate immune responses. However, these inhibitory molecules are exploited by tumor cells to create tumor tolerance and T-cell exhaustion.^4^ To overcome this, anti-CTLA-4, anti-PD-1, and anti-PD-L1 immune checkpoint inhibitors (ICIs) can bind to these coinhibitory receptors, and restore the immune response against tumor cells.^5^

Tasuku Honjo and James Allison received the 2018 Nobel Prize in Physiology for their discoveries in cancer immunology.^6^ Their work led to the development of three groups of ICIs that have been approved by the Food and Drug Administration US (FDA) for the treatment of several cancer types: PD-1 inhibitors (Nivolumab/Pembrolizumab/Cemiplimab), PDL-1 inhibitors (Atezolimumab/Durvalumab/Avelumab), and CTLA-4 inhibitor (Ipilimumab).^7^ Professor Honjo discovered PD-1 on T cells, while Professor Allison discovered another important immunosuppressive molecule: CTLA-4^6^.

Given the intricate nature of tumors and the connection of various genomic and cellular factors in the development and spread of cancer, there is a growing need for the development of effective immunotherapies that can target tumors at both the genetic and cellular levels. One promising approach is chimeric antigen receptor T-cell therapy (CAR-T), which contains engineering T-cells derived from a patient’s blood to express artificial receptors that specifically recognize tumor antigens.^8,9^ The initial step in CAR therapy is leukapheresis, where peripheral blood is isolated from a patient.^9,10^ CAR-T cells can directly identify tumor antigens without relying on the major histocompatibility complex. The application of CAR-T cell treatment in recent years has experienced significant success, by decreased rates of up to 80% in hematological malignancies, particularly in acute lymphoblastic leukemia and non-Hodgkin’s lymphoma.^11^

Monoclonal antibodies (mAbs) are antibodies generated from identical copies of a single B-cell clone, which can attach to specific regions of antigen molecules or epitope.^12^ Moreover, these antibodies can specifically target tumor cells and initiate durable antitumor immune responses. Their multifunctional nature as a therapeutic tool has paved the way for advancing innovative cancer treatment approaches, which are expected to significantly influence cancer care. Therefore, mAbs such as rituximab, cetuximab and trastuzumab, are designed to target specific antigens on cancer cells or immune cells, which can directly destroy cancer cells, block growth signals, or enhance immune responses.^13,14^

Cancer vaccination encompasses various strategies aimed at inducing, enhancing, or directing antitumor immune responses. These methods involve the management of tumor antigens, often in combination with antigen-presenting cells and other immune modulators. Alternatively, directly modulating the tumor itself can also be employed to achieve this objective.^15^ Therapeutic cancer vaccines are designed to improve the immune response to cancer cells and patient outcomes.^16^

**Figure 1:**
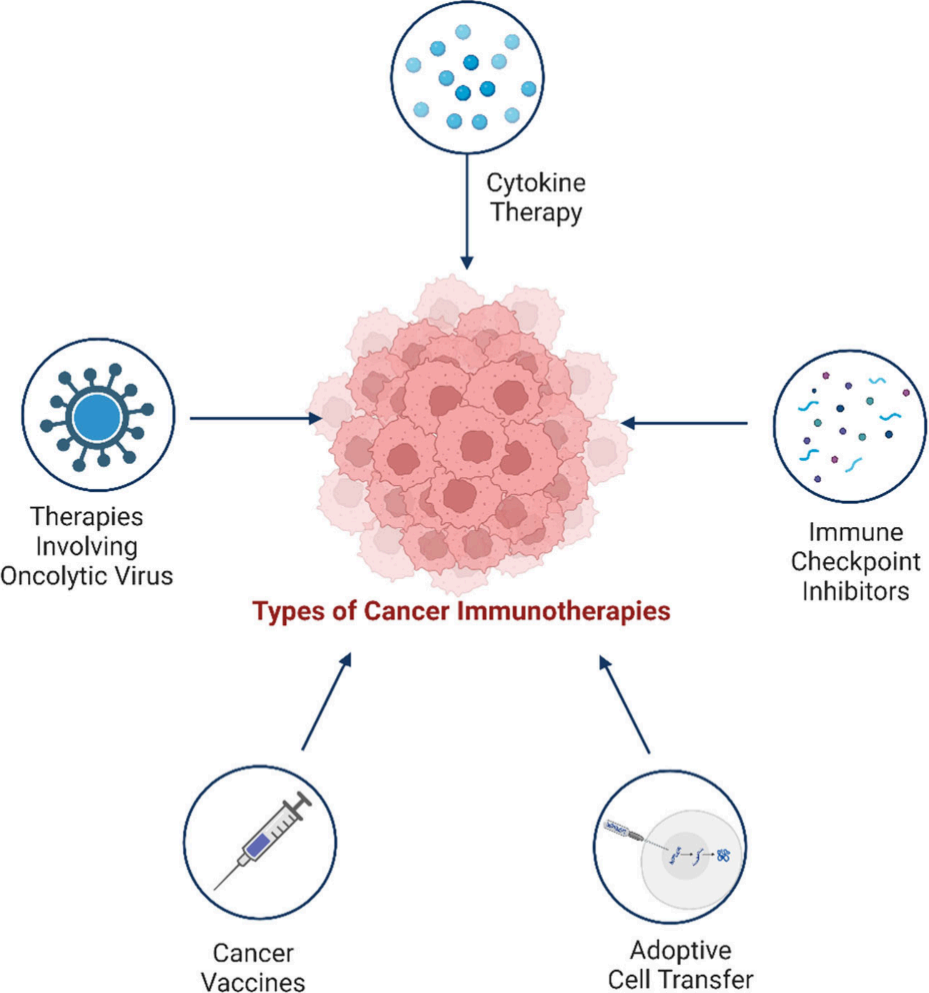
Types of Immunotherapies in the treatment of cancer. Checkpoint Inhibitors: Drugs that unleash the immune system by blocking proteins that prevent it from attacking cancer cells. Monoclonal Antibodies: Laboratory-produced proteins that target specific molecules in cancer cells, marking them for destruction or delivering toxic substances. Adoptive Cell Transfer: Collecting and modifying immune cells in the lab before reintroducing them into the patient’s body to enhance the immune response against cancer. Cancer Vaccines: Stimulate the immune system’s response against cancer cells, either preventing certain types of cancer or targeting existing cancer. Cytokines: Small proteins that activate immune cells and enhance their anticancer activity, used to boost the immune response against cancer. *Oncolytic virus therapy for cancer*: Oncolytic viruses are delivered to the patient. They infect and kill cancer cells, releasing viral particles and tumor antigens. The viral particles infect more cancer cells, while the tumor antigens trigger an immune response. The immune system clears the remaining cancer cells and prevents relapse.

The objective of precision personalized medicine is to adapt treatments to individual patients based on their unique genetic, environmental, and lifestyle factors. In immune-mediated diseases, precision medicine allows for the identification of specific biomarkers and targets, enabling targeted therapies that maximize efficacy and minimize adverse effects.^17^ Advances in genomic sequencing, proteomics, and bioinformatics have facilitated the identification of potential therapeutic targets and personalized treatment strategies for diseases like cancer.^17^ The roadmap toward personalized immunology involves integrating patient-specific data, such as genetic information, immune cell profiles, and environmental factors, to develop individualized immunotherapeutic strategies. This approach enables the identification of optimal treatment options and predictive biomarkers, leading to improved patient outcomes. Advanced technologies, such as high-throughput sequencing and machine learning algorithms, facilitate the development of personalized immunotherapies.^18^Figure 1 shows the main types of immunotherapy methods in cancer treatment. Table 1 gives a comprehensive overview of ICIs approved by the FDA, emphasizing anti-PD-1, anti-CTLA-4, and anti-PD-L1 antibodies, their indications, and their mechanism of action.

**Table 1:** Immune Checkpoint Inhibitors Approved by the FDA for Cancer Immunotherapy and Their Mechanism of Action^211,212^

ICIs	Target	Cancer types	Mechanism of action	Company name
Ipilimumab (Yervoy) (IgG1κ monoclonal antibody-humanized)	CTLA-4	Melanoma, renal cell carcinoma, colorectal cancer, hepatocellular carcinoma	Enhances the activation and expansion of T cells that recognize cancer antigens by blocking CTLA-4	Bristol Myers Squibb
Nivolumab (Opdivo) (IgG4 monoclonal antibody-humanized)	PD-1	Melanoma, nonsmall cell lung cancer, renal cell carcinoma, Hodgkin lymphoma, head and neck cancer, bladder cancer, colorectal cancer, hepatocellular carcinoma, gastric cancer	Restores the function and survival of T cells that are exhausted by chronic exposure to PD-L1 on tumor cells by blocking PD-1	Bristol Myers Squibb
Pembrolizumab (Keytruda) (IgG4 monoclonal antibody-humanized)	PD-1	Melanoma, nonsmall cell lung cancer, head and neck cancer, Hodgkin lymphoma, bladder cancer, gastric cancer, cervical cancer, renal cell carcinoma, hepatocellular carcinoma, Merkel cell carcinoma		Merck & Co.
Cemiplimab (Libtayo) (IgG4 (S228P) kappa monoclonal antibody-humanized)	PD-1	Cutaneous squamous cell carcinoma		Sanofi and Regeneron
Atezolizumab (Tecentriq) (IgG1 monoclonal antibody)	PD-L1	Nonsmall cell lung cancer, bladder cancer, breast cancer	Prevents the inhibition of T cells by PD-L1 on tumor cells or immune cells in the tumor microenvironment by blocking PD-L1	Genentech
Durvalumab (Imfinzi) (IgG1 monoclonal antibody-humanized)	PD-L1	Nonsmall cell lung cancer, bladder cancer		AstraZeneca
Avelumab (Bavencio) (IgG1 monoclonal antibody-humanized)	PD-L1	Merkel cell carcinoma, bladder cancer	Prevents the inhibition of T cells by PD-L1 on tumor cells or immune cells in the tumor microenvironment by blocking PD-L1; also induces antibody-dependent cellular cytotoxicity (ADCC) against tumor cells expressing PD-L1	Pfizer and Merck KGaA

Current immunotherapy, a strategy that enables the immune system of the body to fight tumors, has shown potential in some patients.^19^ However, its effectiveness is often limited by the activation of inhibitory molecules such as CTLA-4 and PD-1, which enable cancer cells to avoid immune detection.^19^ Treatments including immune cytokines, checkpoint inhibitors, and targeted superantigens have been developed, but their success is inconsistent. Agents like interleukin-2 (IL-2) and alpha-interferon (IFN-α) have shown limited effectiveness and extensive toxicity, and many cancer vaccines have not achieved the expected results in clinical trials.^20^

The therapeutic potential of immunotherapy is evaluated using various immune parameters, such as the presence and activation of tumor-infiltrating T cells, PDL1 expression, and tumor mutational burden.^21^ However, the treatment can lead to a range of adverse effects, including autoimmune conditions like thyroiditis and inflammatory bowel disease, and potentially life-threatening events such as myocarditis, encephalitis, and hypophysitis.^19,22^

While immunotherapy has been particularly effective in treating melanoma, its efficacy in other types of cancer is less certain.^19^ Patients generally have a positive attitude toward immunotherapy, but failure to meet expectations can result in significant disappointment, particularly as the majority do not experience the anticipated benefits.^19^ The management of immune-related adverse events is key to enhancing the safety and success of these therapies.^23^

The current approach to overcoming these limitations concerns Targeted Protein Degradation (TPD) or Proteolysis Targeting Chimeras (PROTACs), which involve inducing the selective degradation of proteins. For instance, PROTAC NR-V04 demonstrates rapid and sustained degradation of NR4A1 *in vitro*, with effects lasting up to 4 days in murine models, suggesting potential for enduring therapeutic impact. Its mechanism of action is akin to immune checkpoint inhibitors, a prevalent immunotherapy form, by facilitating the activation of immune cells to target cancer cells.^24^ NR-V04 presents several benefits over conventional antibody-based immunotherapies. It targets intracellular proteins, offering treatment possibilities for patients unresponsive to current immunotherapies. As a small molecule, it can more readily infiltrate the tumor microenvironment. Unlike most immunotherapies that target a single cell type, NR-V04 impacts multiple immune cell types. It has shown excellent safety and efficacy profile in *in vivo*.^24^

This review examines how TPD can modulate the immune system by targeting key regulators of immune responses, such as cytokines, transcription factors, and immune checkpoints. It also addresses the challenges and opportunities of TPD in immunotherapy, such as the optimization of pharmacokinetics and pharmacodynamics (PK/PD), the prediction and mitigation of off-target effects, and the integration of artificial intelligence (AI) to accelerate TPD discovery and development. It concludes by providing insights into TPD’s prospects and directions for immunological interventions.

## 2 PROTAC Technology

Targeted protein degradation is a pharmacological modality based on the induced proximity of an E3 ubiquitin ligase and a target protein to promote target ubiquitination and proteasomal degradation. This has been achieved through PROTACs, bifunctional compounds composed of two separate moieties that individually bind to the target (protein of interest, POI) and the E3 ubiquitin ligase, connected by a linker molecule. By bringing the target protein and the E3 ligase together to form a stable ternary complex, PROTACs induce the ubiquitination and subsequent degradation of the target protein via the proteasome.^25^ This approach has gained attention in drug development as a novel strategy for targeting difficult-to-drug proteins such as those involved in immune disease, cancers, neurodegenerative, and cardiovascular disorders.^26^

PROTAC is an innovation in the medical sciences that has introduced a novel perspective on drug development. PROTACs were developed about 22 years ago. It was first discovered by Sakamoto et al. in 2001 and has evolved from a peptide-based small molecule chimera (Protac-1) to a potential clinical candidate that can be taken orally and that can degrade oncogenic protein.^27,28^

Compared to traditional small-molecule inhibitors, PROTACs provide a novel mechanism by dramatically reducing the accessibility of the targeted POI within cells, thus exhibiting strong selectivity and minimal adverse effects.^29^ PROTACs make use of the natural cell protein degradation mechanism and can degrade specific disease-causing proteins that cannot be targeted with conventional drugs.^30^ In addition to surviving the targeted protein ubiquitination and degradation process, it also preserves its action and participates in multiple future cycles of protein degradation.^31^ This PROTAC event-driven catalytic mechanism of action limits the requirement for a high level of dose availability to a patient, thus eliminating many complications associated with the use of medicinal compounds.^31^

The successful implementation of PROTAC in targeted protein degradation has empowered researchers to move beyond proteasomes. Utilizing the lysosomal degradation pathway, lysosome-targeting chimeras LYTACs accelerate the degradation of extracellular proteins.^32^ Recent advances in the field of autophagy-based degraders, such as AUTAC, ATTEC, MoDE-As, and GalNAc LYTAC, have been successful in degrading several targets via the lysosome.^33^

### 2.1 Overview of PROTACs and Its Mechanism of Action (MoA)

**Figure 2:**
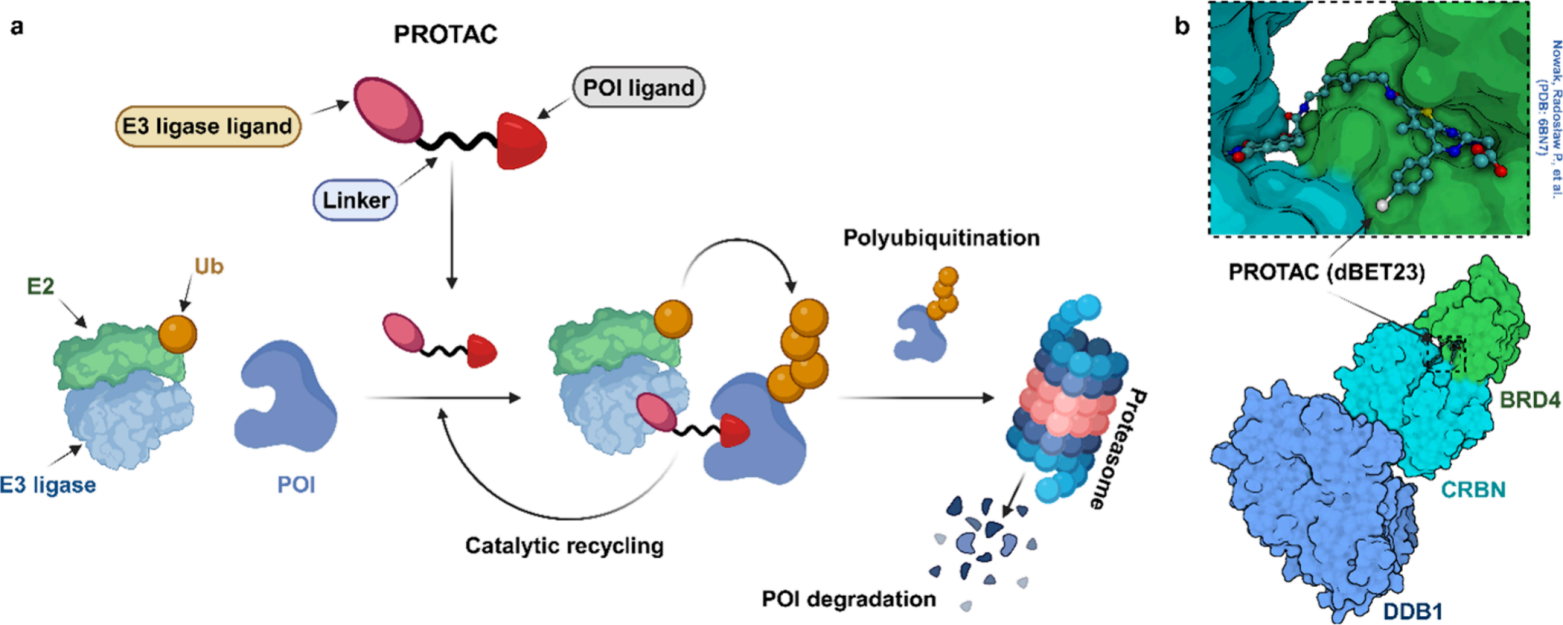
a) The structure and function of PROTACs involve the combination of two ligands: one specific to the POI and the other targeting an E3 ligase. These ligands are connected by a linker, which facilitates the proximity of the POI to the E3 ligase. Subsequently, the target protein undergoes polyubiquitination, where ubiquitin molecules are attached, mediated by an E2 conjugating enzyme. The proteasome then degrades the polyubiquitinated target protein. Notably, the PROTAC itself remains intact throughout this process and can be reused in subsequent cycles, akin to an enzyme’s catalytic cycle. b) Crystal structure-based representation for substrate (BRD4-POI) recruitment to the E3 ligase cereblon (CRBN/DDB1 complex) by a heterobifunctional proteolysis-targeting chimera (PROTAC-dBET23). Ligands are shown as ball and stick representations (Protein Data Bank (PDB) code: 6BN7). DDB1, DNA damage-binding protein 1.

PROTACs are heterobifunctional molecules that contain three components: the protein-of-interest (POI) binding ligand, the variable linker unit, and the E3 ubiquitin ligase ligand (Figure 2). The PROTAC molecule can bind with the E3 ligase and the target protein to form the ternary POI–PROTAC-E3 ligase complex. Hijacking the ubiquitin-protease system (UPS) subsequently causes the target protein to be polyubiquitinated, which is then followed by proteasomal degradation of the protein.^31,34^ In eukaryotic cells, UPS plays a crucial role in maintaining protein homeostasis by eliminating defective and damaged proteins. This system achieves protein degradation through substrate-specific ubiquitination and subsequent recognition. The ubiquitination process involves a coordinated series of enzymatic steps: 1. Ubiquitin Activation (E1): Ubiquitin activating enzymes (E1) activate free ubiquitin (Ub) in an ATP-dependent manner, forming a ubiquitin-E1 thioester bond. 2. Ubiquitin Conjugation (E2): E1 transfers the activated Ub to ubiquitin conjugating enzymes (E2) via transthioesterification. 3. Substrate-Specific Ligases (E3): The Ub-tagged E2, along with the target protein, is recognized by substrate-specific ligases (E3). E3 facilitates the labeling of ubiquitin onto the target protein. 4. Polyubiquitin Chain Formation: These ubiquitination events can be recycled to generate polyubiquitin chains, which serve as tags directing the marked protein to the 26S proteasome for degradation.^31^

In the working mechanism, PROTACs exploit UPS machinery. The initial challenge facing chemical molecules involves crossing the cell membrane (membrane permeability). Specifically, PROTACs exhibit properties that can hinder their permeability. Notably, PROTACs tend to have higher molecular weight and more hydrogen bond donors and acceptors compared to inhibitors. Cellular uptake of PROTACs competes with efflux transporters, a common issue for large molecules. Despite these challenges, many PROTACs efficiently enter cells and achieve concentrations sufficient for their intended activity. When it enters the cell, a PROTAC establishes a binary interaction (either reversible or irreversible) with one of its target proteins. Subsequently, this binary complex recruits the other target protein to form a ternary complex (1:1:1). The significance of this ternary complex lies in the induced proximity between the target protein and the E3 ligase. In the absence of the PROTAC, such specific protein–protein interactions would not naturally occur. Within this complex, the E3 ligase transfers ubiquitin molecules to the target protein. Remarkably, multiple ubiquitin molecules are sequentially added to various sites, forming polyubiquitin chains. Although deubiquitinases (DUBs) exist and can remove ubiquitin from target proteins, this process does not inhibit PROTAC-mediated targeted protein degradation. Polyubiquitylation is a crucial cellular process that plays a central role in maintaining protein homeostasis. This process involves the covalent attachment of multiple ubiquitin molecules to a target protein. Ubiquitylation serves as a signal for protein degradation by directing the tagged protein to the proteasome, where it undergoes controlled breakdown (Figure 2).^35^

### 2.2 Recent Developments on PROTAC

The field of PROTACs has seen remarkable advancements, leading to the development of various types of these innovative molecules with enhanced therapeutic potential.^36−43^ Different types of PROTACs have emerged, each designed to address specific challenges and provide unique advantages in targeted protein degradation. These include ternary PROTACs, photo-PROTACs, homo-PROTAC, SNIPER, Trim-away, and HyT etc.^36−43^ Each type offers distinct features and capabilities, expanding the possibilities for precise and controlled protein modulation in therapeutic applications.^44^Table 2 provides a overview of the different types of PROTAC.^36−43^

**Table 2:** Different Types of Currently Available PROTACs

Type	Definition	Example	Target protein	E3 ligase or degradation pathway	References
Binary PROTAC	A heterobifunctional molecule composed of two ligands connected by a linker: one that binds to the target protein and one that binds to an E3 ligase.	ARV-110	Androgen receptor (AR)	VHL	(39,40)
Ternary PROTAC	A trivalent PROTAC consisted of two POI ligand and an E3 ligase ligand, each separately tethered via a branched linker.	SIM1	BRD2 (BD1 and BD2)	VHL	(38)
Photo-PROTAC	A PROTAC that requires light activation to induce protein degradation.	SPN pro	IDO (indoleamine 2,3-dioxygenase)	VHL and semiconducting polymer nanoparticle	(43)
Homo-PROTAC	A PROTAC that consists of two identical ligands connected by a linker: both ligands bind to the same target protein.	Compound 15a	CRBN	CRBN	(42)
SNIPER (specific and nongenetic IAP-dependent protein eraser)	A heterobifunctional molecule composed of two ligands connected by a linker: one that binds to the target protein and one that binds to an IAP (inhibitor of apoptosis protein).	SNIPER(ER)-87	Estrogen receptor alpha	XIAP or cIAP1	(41)
Trim-away	E3 ligase TRIM21 to recruit antibody-bound pathogens or proteopathic agents by using antibodies against a protein of interest for subsequent ubiquitin-mediated target degradation	Trim-Away	Kinesin-5 (Eg5)	TRIM21	(36)
Hydrophobic tag (HyT)	HyTs, consisting of a POI ligand linked to a hydrophobic degron that mimics the exposed hydrophobic region of misfolded proteins and is recognized by HSP70.	HyT 3	mutant huntingtin (mHTT)	HSP70 mediated proteasomal degradation	(37)

In particular, several promising PROTACs are currently undergoing preclinical and clinical evaluations.^28^ Some examples of progress in preclinical studies include investigating phototherapeutic semiconducting polymer nano-PROTAC for activatable photoimmunometabolic cancer treatment, developing carbon dots-based PROTACs by specifically activating the STING pathway that leads to degradation of PD-L1, along with the fabrication of PROTAC-induced BET for prostate cancer.^43,45,46^ Recently, researchers have concentrated their efforts on designing novel ligands with increased selectivity for specific target proteins.^34^ The capability of PROTACs to specifically degrade target proteins offers a novel avenue for addressing diseases that pose challenges to established small-molecule drugs. With ongoing advancements in the field of PROTAC technology, the development of new types and optimization strategies of PROTAC will improve and broaden the possibilities of this highly promising drug class. One of the challenges encountered during the development of PROTACs is the optimization of the PK/PD properties. Like other small molecule drugs, PROTACs should be able to achieve their target proteins *in vivo* and induce degradation while maintaining appropriate pharmacokinetic parameters such as half-life, clearance, and bioavailability. Furthermore, selective degradation of target proteins can lead to off-target effects, which must be carefully monitored and controlled.^47^

In order to meet these challenges, researchers are exploring new approaches to optimize PROTAC. As an example, a new technology report outlined the development of the “halo-PROTAC” strategy, which involves the incorporation of a haloalkane moiety into the ligand linker. This moiety can be used for the selective modification of PROTAC by fluorine-18, enabling the imaging and monitoring of the drug *in vivo*.^48,49^ Another approach for the optimization of PROTACs involves the use of artificial intelligence (AI) and machine learning (ML). This enables researchers to identify new ligand structures and predict their pharmacological properties by analyzing large data sets of molecular structures and properties. This approach was used to predict the binding affinity of PROTACs to their target proteins and to design new ligands with greater potency and selectivity.^50,51^

### 2.3 Applications of Protein Degraders for Targeted Therapy

Protein degraders find significant applications in cancer therapy, with over ten TPD molecules currently undergoing clinical trials.^52,53^ Beyond cancer, these TPD molecules hold substantial potential for treating neurodegenerative diseases, inflammatory conditions, and viral infections.^54^ Conventional cancer therapies, often suffer from limitations in selectivity, leading to significant side effects due to their nonspecific toxicity toward healthy cells. In contrast, protein degraders offer a targeted approach by selectively breaking down oncogenic proteins critical for cancer cell survival and progression while sparing normal cells. Notably, kinases constitute 45% of the total targets degraded by PROTACs.^31^ Among these, more than half of the PROTACs specifically target receptor tyrosine kinases (RTKs).^31^ For example, PROTACs designed to degrade proteins like BRD4, BCR-ABL, and ERs have shown promising results in both preclinical and clinical studies. These findings underscore the potential of protein degradation as an innovative strategy for treating various cancer types, including breast cancer, leukemia, and prostate cancer. Neurodegenerative diseases, including Alzheimer’s, Parkinson’s, and Huntington’s, are characterized by the accumulation of misfolded proteins in the brain, leading to cognitive dysfunction and mobility impairment. Conventional small-molecule agents struggle to modulate these protein aggregates, making them challenging drug targets. However, recent advances in targeted protein degradation offer hope. Bifunctional molecules, such as PROTACs, recruit disease-related proteins to cellular degradation pathways like the ubiquitin-proteasome system and autophagy-lysosome pathway. Notably, PROTACs designed to target proteins like tau, alpha-synuclein, and huntingtin have demonstrated promising results in preclinical models.^55^ These findings highlight the potential of protein degraders as a therapeutic strategy for neurodegenerative disorders. Further details on PROTAC applications are discussed in Section 4.

### 2.4 Targeted Protein Degradation by Proteasomal vs Lysosomal Pathway

Protein degradation is an important cellular process that maintains protein homeostasis and regulates cellular functions.^56^ Proteasomal and lysosomal degradation pathways are two key mechanisms by which cells selectively degrade specific proteins in cells.^57^ These pathways play an essential role in various cell processes, including cell cycle regulation, signal transmission, protein quality control, and apoptosis.^56^ They also modulate the immune system and the antitumor response, by affecting the antigen presentation, the expression of immune checkpoints, and the activation of immune cells. Understanding the differences between proteasomal and lysosomal degradation pathways is important for gaining insights into their distinct functions and potential therapeutic applications.^58^ For example, TPD technologies, such as PROTAC and lysosomal targeting molecules, can exploit these pathways to selectively degrade cancer-associated proteins and enhance the efficacy of immunotherapy.

The proteasome is a large protease complex that serves as the main proteolytic machinery in the cell for selective protein degradation.^59^ The proteasomal degradation pathway includes the UPS, which labels proteins with ubiquitin molecules and targets them for degradation by the proteasome. The UPS shows a crucial role in the degradation of short-lived regulation proteins, defective or damaged proteins, and proteins involved in the progression of the cell cycle.^58^

The 2004 Nobel Prize for Chemistry was awarded jointly to Aaron Ciechanover, Avram Hershko and Irwin Rose “for the discovery of ubiquitin-mediated protein degradation”, which revealed the molecular mechanisms, and biological significance of the proteasome and its regulation.^60^ The proteasomal-based degradation discussed in Section 2.1.

Lysosomes are membrane-bound organelles that serve as the main site for intracellular degradation of cell components, including proteins, lipids, and carbohydrates.^61^ The lysosomal degradation pathway involves the lysosome, which contains various hydrolytic enzymes capable of breaking down different type of macromolecules.^62^ In the lysosomal degradation pathway, cell components are engulfed by membrane vesicles called autophagosomes that then fuse with the lysosomes to form the autolysosomes. The content of the autolysosomes is then degraded by lysosomal enzymes, such as proteases, lipases, and glycosidases, resulting in the breakdown of cellular components into their constituent molecules for recycling or disposal. Ghosh et al. (2022) extensively reviewed the lysosomal pathway for targeted protein degradation.^33^

### 2.5 PROTAC Drug Research Evaluation and Its Various Clinical Trial Status

The PROTACs sector has experienced significant growth in recent years.^53^ In terms of clinical development, PROTACs have shown promising results. Arvinas, Inc. has achieved successful development of the first two PROTAC degraders: Bavdegalutamide (ARV-110) and Vepdegestrant (ARV-471). These compounds specifically target androgen receptors for prostate cancer treatment and the estrogen receptor for breast cancer. Currently, they are undergoing phase II and III clinical trials, respectively (FDA clinical trial number NCT05909397).^63^

Several PROTACs are currently in clinical development, targeting a diverse array of proteins.^64^ Notable targets include androgen receptor (AR) with compounds such as CC-94676, ARV-110, and ARV-766, as well as estrogen receptor (ER) with AC682 and ARV-471. Additionally, BRD9 is addressed by FHD-609 and CFT8634, while BCL-xL is targeted by DT2216. Other notable targets include IRAK4 (KT-474 and KT413), STAT3 (KT-333), BTK (NX-5948 and NX-2127), TRK (CG001419) and EGFR-L858R mutant (CFT8919). These PROTACs hold promise across indications including hematological malignancies, solid tumors, synovial sarcomas, and autoimmune diseases (Table 3).^64,65^

**Table 3:** Summary of PROTACs in Clinical Trials^64,65^

PROTACs	Target protein	E3 ligase	Phase	Company	Indication
ARV-471	ER	CRBN	III	Arvinas	Breast cancer
ARV-110	AR	CRBN	II	Arvinas	Prostate cancer
ARV-766	AR	CRBN	I	Arvinas	Prostate cancer
CC-94676	AR	CRBN	I	Bristol Myers, Sqibb	Prostate cancer
DT2216	BCL-XL	VHL	I	Dialectic	T cell lymphomas
FHD-609	BRD9	-	I	Foghorn	Synovial sarcoma
CFT-8634	BRD9	CRBN	I	C4 Therapeutics	Synovial sarcoma
NX-2127	BTK, IKZF1/3	CRBN	I	Nurix	B- cell malignancies
BGB-16673	BTK	NA	I	BeiGene	B-cell malignancies
NX-5948	BTK	CRBN	I	Nurix	B-cell malignancies and autoimmune diseases
HSK29116	BTK	NA	I	Haisco	B-cell malignancies
KT-474	IRAK4	CRBN	I	Kymera	Immuno-inflammatory skin disease
KT-413	IRAK4, IKZF1/3	CRBN	I	Kymera	MYD88 mutant tumors
KT-333	STAT3	NA	I	Kymera	Liquid and solid tumors, T cell lymphomas
CG001419	TRK	CRBN	I	Cullgen	-
AC-0176	AR	CRBN	I	Accutar	Prostate cancer
HP518	AR	CRBN	I	Hinova	Prostate cancer
GT20029	AR	CRBN	I	Kintor	Androgenetic alopecia and acne vulgaris
CFT-1946	BRAF V600	CRBN	I	C4 Therapeutics	BRAF V600 mutant solid tumors, nonsmall-cell lung cancer, colorectal cancer and melanoma

In brief, The BTK PROTAC NX-2127 effectively degrades both wild-type and C481S mutant BTK, surpassing ibrutinib in xenograft mouse models. NX-2127 achieved 80% BTK degradation, including resistant mutations (IKZF1/3 degradation observed). In contrast, PROTAC NX-5948 selectively degrades BTK without affecting IKZF1/3, avoiding immunomodulatory effects.^65,66^

The PROTAC approach, despite its potential to target traditionally “undruggable” proteins, often focuses on already well-characterized targets with high-quality ligands. These ligands aid in designing heterobifunctional candidate drugs. While preclinical evidence supports degradation over inhibition, clinical data validation is crucial. The STAT3 degrader KT-333 represents a significant advancement, as STAT3 is challenging to drug conventionally. KT-333 exhibits potent apoptotic and antiproliferative effects *in vitro* and in lymphoma models when administered intravenously.^65,67^

PROTAC KT-474 stands out as one of the rare degraders currently in trials for noncancer indications. It targets Interleukin-1 receptor-associated kinase 4 (IRAK4), which transduces signals from toll-like receptors via myeloid differentiation primary-response protein 88 (MYD88). Loss-of-function mutations in either protein lead to similar immune deficiencies. While IRAK4 kinase inhibitors are being tested for inflammatory diseases, the protein’s scaffolding functions also play a role in immune signaling. KT-474 degrades IRAK4 and suppresses proinflammatory gene expression in the skin of patients with hidradenitis suppurativa and atopic dermatitis, administered orally.^65,68^

## 3 Why Such an Interest in Immunology?

There is growing interest in cancer immunotherapy, which intersects with the developing field of TPD due to the potential synergy and enhanced treatment outcomes they offer.^69,70^ The main reasons for the interest in the combination of cancer immunotherapy and TPD:

### 3.1 Overcoming Resistance Mechanisms

Cancer cells often exploit resistance to targeted therapies, leading to treatment failure. TPD offers an innovative method to address resistance by selectively degrading disease-causing proteins, including those involved in cancer progression and immune evasion. By combining this approach with immunotherapy, which activates and utilizes the immune system to target cancer cells, the dual mechanism can potentially overcome resistance and enhance treatment effectiveness.^71^

### 3.2 Complementary Mechanisms of Action

Cancer immunotherapy primarily focuses on activating and boosting the body’s immune response to cancer cells. Targeted protein degradation, on the other hand, selectively eliminates specific disease-causing proteins that contribute to tumor growth and survival. By combining these approaches, the immune system can support the recognition and targeting of cancer cells while removing key proteins that support tumor growth and immune escape while preventing the immune system from being compromised. Immune systems can increase the recognition and targeting of cancer cells and at the same time eliminate key proteins supporting tumor growth and immune deterrence, leading to a more comprehensive and targeted attack against cancer.^72^

### 3.3 Enhanced Tumor Antigen Presentation and Selectivity

Targeted protein degradation can modulate the expression of proteins involved in antigen presentation, a crucial step in activating the immune system against cancer cells. By selectively degrading proteins that suppress antigen presentation, immunotherapy can be potentiated, resulting in improved recognition of cancer cells by immune cells and a more robust antitumor immune response. Jensen et al. demonstrated the impact of targeted protein degradation on antigen presentation.^73^ Specifically, they investigated the production of major histocompatibility complex class I (MHC-I) specific peptides following the degradation of bromodomain proteins using BET PROTACs. These PROTACs were designed for CRBN, VHL, and MDM2 ligases and were employed to degrade BRD2, BRD3, and BRD4. Post-treatment, the resulting MHC-I complexes were isolated and analyzed via LC-MS. The study revealed that PROTACs facilitate the display of BRD2/3/4 peptides on the cell surface, potentially enhancing targeted immunotherapy.^73^

**Figure 3:**
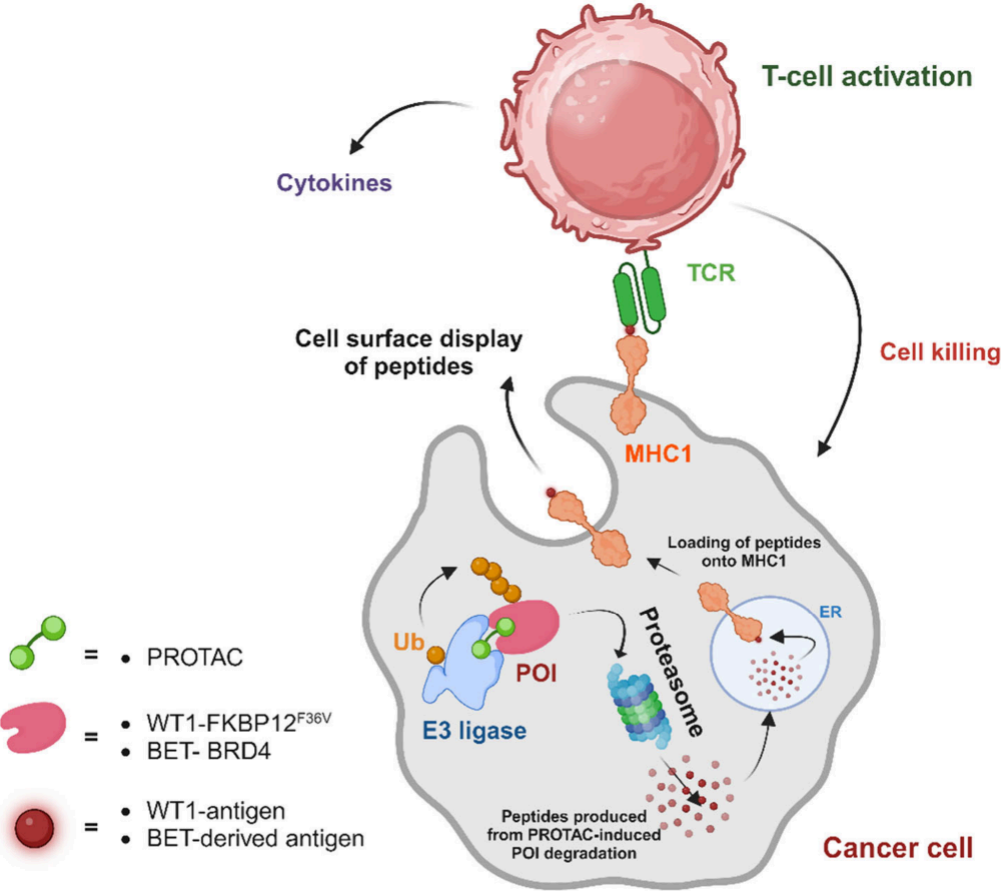
PROTAC-mediated modulation of the immune response against cancer cells. Tumor cell killing is selectively carried out by T cells, which recognize T cell receptor (TCR) antigens produced from PROTAC-induced proteolysis. The cell surface displays new MHC-I peptides derived from cancer cell-specific antigens via PROTAC-induced protein degradation. PROTACs specifically target proteins expressed in cancer settings, resulting in the generation of unique MHC-I complexes. These complexes can be recognized by TCRs on T cells. The proteins of interest (POIs), WT1 and BET, have confirmed protein peptides that serve as surface antigens for T cell recognition.^34,73,74^

Furthermore, this approach was extended to induce the display of peptides from a model antigen, GFP-S8L-F12, using a dTAG-7 system in macrophage (BMC-2) and dendritic (DC2.4) cell lines.^74^ The research emphasized the importance of proteasomal cleavage of mature proteins in generating MHC-I antigens, as opposed to short-lived defective ribosomal products. Additionally, the study hinted at the possibility of synergizing PROTACs with other strategies to amplify direct MHC class I presentation (Figure 3a).^74^

To achieve tissue selectivity, consider the example of the BRDT protein—a cancer-testis specific bromodomain protein frequently expressed in lung cancer. By degrading BRDT using PROTACs, its peptides can be displayed on the cell surface, providing a unique opportunity for selective immune cell targeting of BRDT-expressing tumors.^34^ Importantly, this selectivity arises from the peptides produced through proteasomal degradation rather than the PROTAC molecules themselves. These insights pave the way for leveraging differential peptide processing induced by PROTACs to enhance the precision of immunotherapeutic interventions.^34^

The initial confirmation of PROTAC degradation of BRD4 increases the presentation of antigenic BRD4 peptides displayed on MHC I.^73,74^ The proximity of the E3 ligase to the target protein plays an essential role in degrader activity on antigen presentation. Subsequent studies delved into more detailed targeting, focusing on Wilms tumor 1 (WT1).^75^ Degrader molecules, such as the PROTAC RMF-TCB, enhance the antitumor immune response. Degradation of WT1 by RMF-TCB increases the percentage of T cells expressing the early activation marker CD69 and the percentage of CD8+ T cells expressing the late activation marker CD25.^75^ Additionally, PROTAC treatment leads to increased cytokine secretion and enhanced tumor-killing activity of effector CD8+ T cells. Targeted protein degradation in cancer cells can activate T cells and improve effector function, offering potential for future modulation of antigen-specific immune responses (Figure 3).^75^

### 3.4 CAR T Cell Controlled and Enhanced by PROTAC

**Figure 4:**
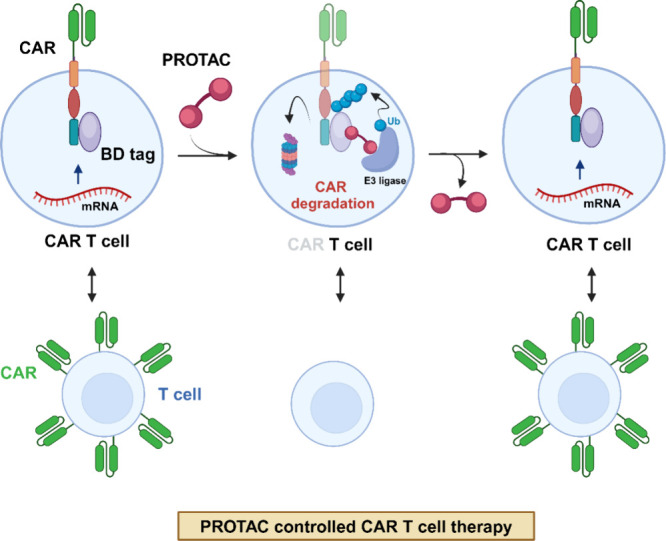
Model illustrating the control of CAR T cell activity through CAR degradation. A novel CAR T cell safety strategy specifically targets the CAR protein rather than the CAR T cell itself. The PROTAC compound, directed against the bromodomain (BD), degrades the BD-containing CAR protein. Notably, CAR expression is restored upon removal of the PROTAC compound from the cell or system, demonstrating its reversibility.^76^

The application of CAR (chimeric antigen receptor) T cell therapy has been associated with significant safety concerns, including fatalities during clinical trials. Lee et al. have demonstrated a successful proof-of-concept for CAR degradation.^76^ The engineered CAR PROTAC molecule effectively inhibited the lytic function of CAR-T cells through the degradation of CAR proteins (Figure 4). The proposed PROTAC-based CAR T cell safety strategy, which targets the CAR protein, not the CAR T cell. This strategy is advantageous for the controlled, reversible activation of CAR T cells, thereby mitigating immune-related toxicity.^76^

### 3.5 Synergistic Effects on the Tumor Microenvironment

Both cancer immunotherapy and targeted protein degradation have the potential to modulate the tumor microenvironment, which plays a significant role in tumor growth and immune response. Targeted protein degradation can alter the composition and signaling within the tumor microenvironment, making it more favorable for immune cell infiltration and activation. This, in turn, can enhance the effectiveness of immunotherapy by creating a more conducive environment for immune-mediated tumor destruction.^77,78^

### 3.6 Personalized and Combination Therapies

TPD and cancer immunotherapy can be tailored to individual patients based on their specific genetic and molecular characteristics. Biomarkers and molecular diagnostics can help identify patients who may benefit from these therapies and provide personalized treatment strategies. Furthermore, the combination of TPD and immunotherapy allows for the possibility of customized treatment regimens that address specific vulnerabilities and immune response characteristics of each patient’s tumor.^79,80^

### 3.7 Potential for Novel Therapeutic Targets

The field of TPD offers a versatile platform for identifying and selectively degrading disease-causing proteins, including previously “undruggable” targets (STAT3, KRAS^G12C^, and CDK2).^81^ This opens new possibilities for combination therapies with immunotherapy, as novel targets can be used further to strengthen the immune response to cancer cells.^82^

The interest in combining cancer immunotherapy and targeted protein degradation arises from the potential synergistic effects and the ability to address challenges such as resistance mechanisms, tumor heterogeneity, and immunosuppressive microenvironments. By leveraging the strengths of both approaches, researchers and clinicians aim to achieve improved treatment outcomes and ultimately provide more effective and durable therapies for cancer patients.^64^

## 4 PROTACs in Immunotherapy

**Figure 5:**
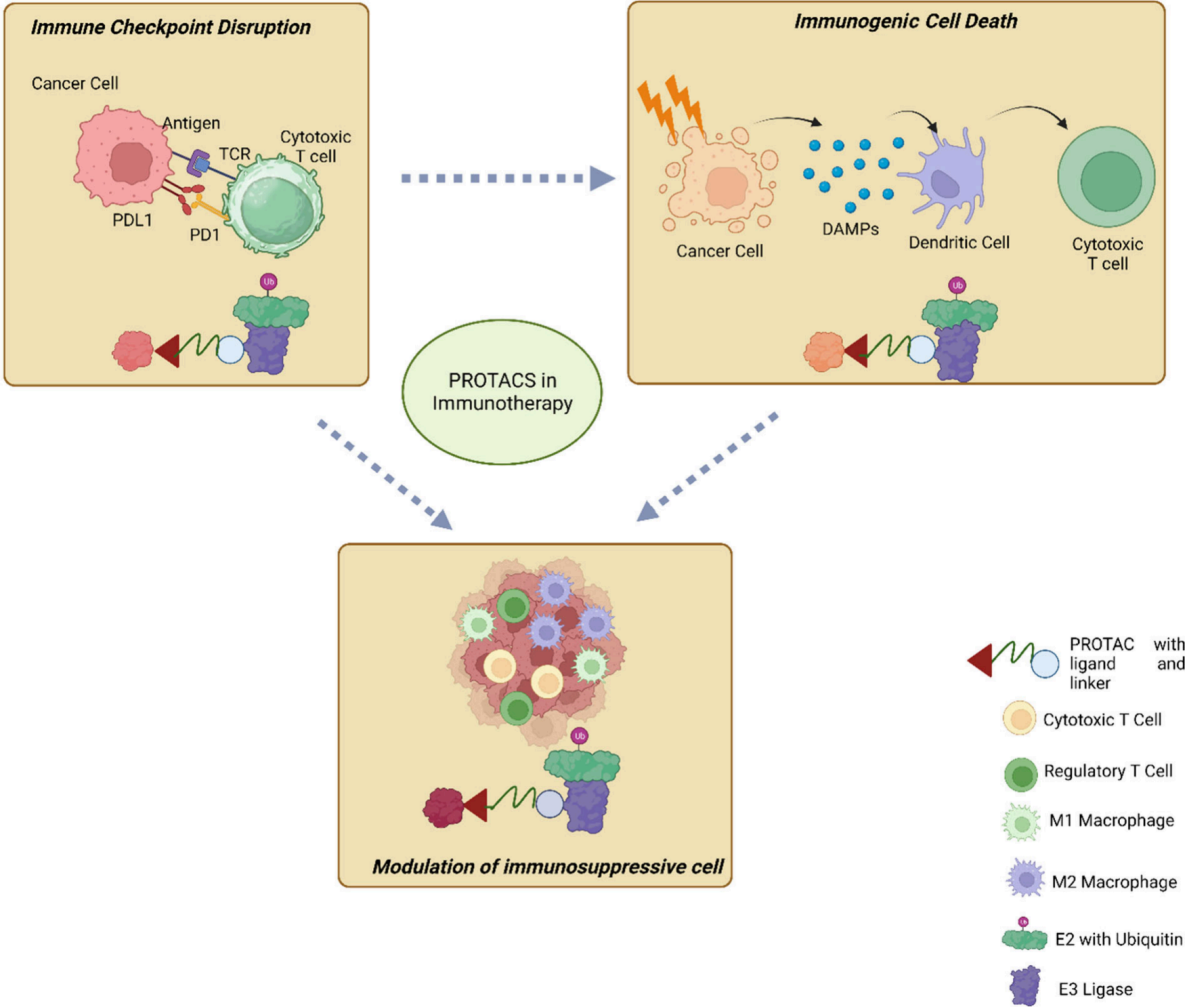
PROTAC-based cancer immunotherapy aims to transform the immunosuppressive tumor microenvironment into an immunoactive state through three distinct pathways. First, the application of PROTACs leads to the elimination of oncogenic proteins that are crucial for the growth and survival of cancer cells, thereby inducing immunogenic cell death. Second, PROTACs disrupt the immune checkpoint present on cancer cells, rendering them susceptible to immune attack by cytotoxic T cells. Lastly, PROTACs effectively eradicate immunosuppressive signal-associated cytokines, thus reducing the population of regulatory immune cells within tumor tissues.

PROTAC is a new type of drug that has been studied to improve immunotherapy.^69,78^ PROTACs work by targeting and degrading proteins that are essential for cancer cell survival. This can cause cancer cells to die without harming healthy cells. Immunotherapy and chemotherapy are commonly used as first-line treatment methods but have several limitations and drawbacks.^19,83,84^ These include limited therapeutic benefits, the potential for serious adverse side effects unrelated to the intended target, the long half-life of drugs, poor oral bioavailability, drug resistance, and challenges in targeting specific proteins.^19,83,84^ PROTACs, however, are emerging as a promising solution to these challenges.^31,85^ Until now, PROTAC has been used to treat many immunological disorders.^78^ There are some advantages of using PROTACs such as eliminating pathogenic proteins, eliminating active sites, targeting undruggable and intracellular targets, penetrating tissue, providing systemic delivery, it is having a catalytic mode of action. Figure 5 depicts the possible utilization of PROTACs in cancer immunotherapy.

### 4.1 Significant Discoveries and Advances in the Field of PROTAC Immunotherapy

Recently, there have been significant advancements in immunotherapy using PROTACs to target various proteins, including Bcl-xL (B-cell lymphoma extra-large), BET (bromo- and extra terminal)/FKBP12 (FK506-binding protein 12), COX-1/2 (cyclooxygenase-1 and 2), HDAC (histone deacetylases), H-PGDS (hematopoietic prostaglandin D Synthase), IDO1 (indoleamine 2,3-dioxygenase 1), IRAK (Interleukin-1 receptor-associated kinase), JAK (Janus kinase), NAMPT (nicotinamide phosphoribosyl transferase), PD-L1 (programmed cell death-ligand 1), SHP2 (src homology-2 domain-containing protein tyrosine phosphatase), SIRT2 (sirtuin 2), and STAT3 (signal transducer and activator of transcription 3) are discussed here.

#### 4.1.1 PD-L1 (Programmed Cell Death-Ligand 1) PROTACs

T cells express PD-1 proteins on their surfaces and react with PD-L1 ligands expressed in tumor cells.^86^ It is known that PD-L1, a regulatory molecule, has an immunoregulatory function that decreases the excessive immune response when it binds to its ligand. The mechanism of the PD-1/PD-L1 pathway is to inhibit excessive tissue destruction during inflammatory disease and act as an immune checkpoint.^87^ Some cancer cells exploit this checkpoint to bypass the immune response against it. One of the more advanced and promising immunotherapy strategies is the inhibition interaction between PD-1 and PD-L1.^88^ Studies show that PROTAC molecules can be used as an effective treatment against cancer.^85^ PROTAC 21 has shown that it effectively improved the degradation of PD-L1 in many cancer cells by the proteasome. However, it was also shown that 21a effectively decreased PD-L1 expression levels of MC-38 malignant cells *in vivo* which induced invasion of CD8+ T cells which in turn prevented the tumor growth of MC-38 *in vivo*.^89^

**Figure 6:**
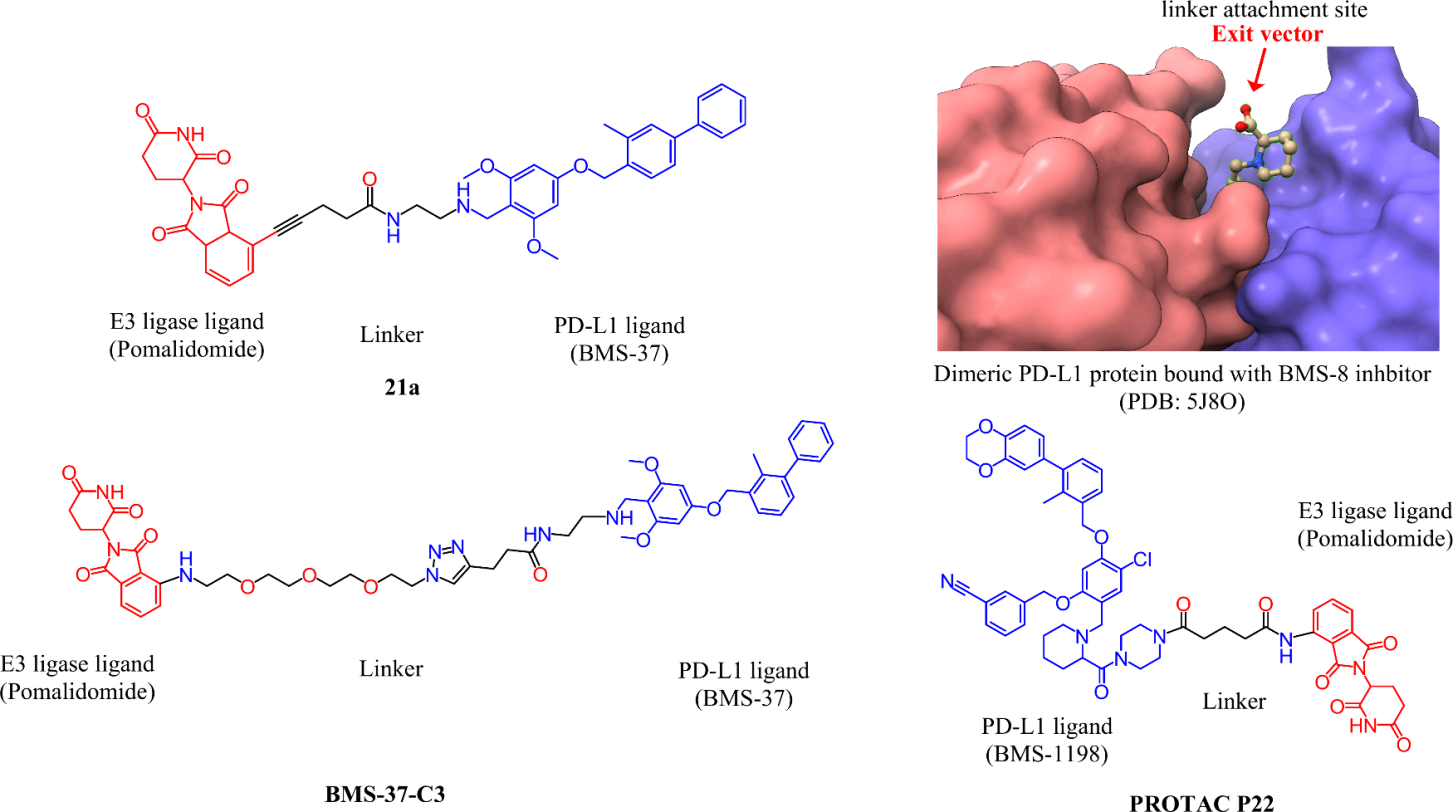
Chemical structures of PD-L1 degraders. The figure shows the chemical structures of three PD-L1 degraders: 21a, BMS-37-C3, and P22. The structures are drawn using ChemDraw. In the top right corner, a 3D representation of a PD-L1 bound BMS-8 inhibitor is shown. Red arrows show the optimal linker attachment sites.

As an example, the recent synthesis of biphenyl BMS-37 (PD-L1 inhibitor) based PD-L1 degraders targeted different E3 ligases, such as von Hippel-Lindau (VHL), Cereblon (CRBN), Mouse double minute 2 homologue (MDM2), or Cellular inhibitor of apoptosis (cIAP). Among these compounds, the one that used the CRBN ligand BMS-37-C3 was the most effective in degrading PD-L1. The BMS-37-C3 molecules also improved the ability of T-cells to kill A375 cells in a coculture model, compared to Atezolizumab anti-PD-L1 antibody.^90^ These results indicate that synthetic PROTACs could lead to a novel therapeutic method for tumor immunotherapy, particularly in the case of melanoma.^90^ Another compound that showed promising activity was P22, which had BMS-1198 attached to pomalidomide as the CRBN E3 ligase ligand with a piperazine linker.^91^ P22 showed superior inhibitory activity than the rest of the series, with an IC_50_ value of 39.2 nM. The activity of P22 was measured by HTRF binding assay and further confirmed by FACS, Western blot, and biochemical assays. In the study, authors synthesized 28 compounds with different linker lengths and found that rigid piperazine linkers were more effective in inhibiting PD-1/PD-L1 than flexible or straight linkers. These findings indicate that the choice of linker is important for designing potent and selective PD-L1 degraders.^91^ Some of the PD-L1 degraders that are under development have their chemical structures illustrated in Figure 6. These compounds are designed to bind to PD-L1 and either promote its internalization or induce its degradation by the proteasome.

Another approach to designing PD-L1 degraders is to use stapled peptides that mimic PROTACs. A recent study reported on a PROTAC stapled peptide specifically targeting ZDHHC3, a palmitoyltransferase that regulates PD-L1 stability.^92^ This stapled peptide PROTAC (SP-PROTAC) was more effective than BMS-8, a PD-L1 inhibitor, in reducing PD-L1 levels and enhancing cytokine production and T-cell activation in human cervical cancer cells.^93^ Further, this study showed how SP-PROTACs can act as novel dual agents that both inhibit and degrade PD-L1. Recently, a novel system has been described using peptides targeting PD-L1 and PD-1 linked to E3 ligase/protein binding components to induce their degradation. This peptide-PROTAC system can effectively lower the PD-L1 and PD-1 expression in cervical cancer cells and boost the immune system’s ability to fight tumors. This study shows that peptides can be used as an alternative strategy to design potent and selective PD-L1 and PD-1 degraders.^93^

Recent reports have also been made of a novel PROTAC that used carbon dots (CDs) as scaffolds to degrade PD-L1 protein and activate the STING pathway in tumor cells.^46^ These CD-based PROTACs (CDTACs) can be combined with PD-L1, recruit CRBN, induce PD-L1 ubiquitination, and degrade them by proteasomes. CDTACs in CT26 or B16–F10 tumor cells can degrade more than 99% or 90% of PD-L1. In addition, CDTACs can activate the STING pathway to trigger immune reactions.^46^ These results indicate that CDTACs are a promising type of PROTACs that can degrade membrane proteins and modulate immune pathways.^46^

Antibody-based PROTACs (AbTACs) are a new type of PROTACs that use antibodies to target and degrade cell-surface proteins, such as PD-L1, which are hard to reach with conventional PROTACs.^94^ AbTACs can activate the STING pathway and may be useful for cancer treatment. AC-1 is an AbTAC that uses RNF43, a cell surface E3 ligase, to degrade PD-L1, which does not have a small molecule ligase. PD-L1 is degraded in the lysosomes by AC-1, which does not interfere with other proteins. AC-1 has a D_Max_ of 63%, which is the balance between the rate of synthesis and degradation.^94^

#### 4.1.2 IDO (Indoleamine 2,3-dioxygenase) PROTACs

IDO is an enzyme that prevents immune response against tumors.^95^ This enzyme functions as immunosuppressive through tryptophan metabolism or nonenzymatic function. Inhibitors of this enzyme act by preventing tryptophan metabolism.^95^ However, these drugs have not succeeded in enhancing the survival rate of cancer patients,^96^ so a new emerging approach to degrade IDO1 is PROTAC.^97^ PROTAC targeting IDOC degrades this protein by proteasome IDO1 converts tryptophan to kynurenines many inhibitor drugs were used against IDO1 but failed to inhibit its nonenzymatic immunosuppressive functions. It is very common in several cancers that IDO1 suppresses the immune response against tumors.^95^ Some small molecules that can block both the enzymatic and nonenzymatic activities of IDO1 are being developed.^96^

IDO1 is a challenging protein to drug, yet it plays a crucial role as a target in cancer immunotherapy. To address this, researchers have developed PROTAC, a powerful and effective method for selectively degrading IDO1. The first IDO1 PROTAC compound, known as 2c, was designed by conjugating the established IDO1 inhibitor epacadostat with the CRBN (Cereblon) ligand pomalidomide.^97^ In HeLa cells, 2c demonstrated remarkable and sustained degradation of IDO1, achieving a maximum degradation (Dmax) of 93%. Furthermore, 2c exhibited moderate enhancement of HER2 CAR-T cell activity.^97^

In the search of PROTAC design, researchers have also explored the use of the enzyme inhibitor BMS986205 against IDO1 binding ligands.^98^ Analogies of BMS986205 is BMS 116 which has the advantage of a more accessible phenyl group toward solvent.^98^ IDO1-PROTAC is designed and synthesized by connecting several linker groups to the phenyl groups of BMS-986205 molecules. IDO1-PROTAC was able to inhibit IDO1 activity in human brain cancer cells (glioblastoma) in culture and reduced IDO1 protein levels in brain tumors in animals.^98^ This innovative approach holds promise for advancing cancer therapeutics by specifically targeting challenging proteins like IDO1.

#### 4.1.3 BTK (Bruton Tyrosine Kinase) PROTACs

BTK plays a key role in signaling the antigen receptors of B cells. It regulates various processes, such as proliferation, maturation, and programming cell death of B cells.^99^ In recent years, inhibiting BTK has become an effective therapeutic approach for treating hematological malignancies and autoimmune diseases.^100,101^ One innovative method for targeted treatment of BTK-related diseases involves using PROTAC technology.^102^ The research conducted by Buhimschi’s team resulted in the creation of MT-802, a PROTAC that effectively degrades wild-type and C481s mutant BTK.^103^ This was achieved by utilizing BTK-specific and CEBN-specific ligands to recruit BTK to the E3 ligase complex, directing its degradation through the proteasome. Compared to Ibrutinib, MT-802 demonstrated high potency in degrading BTK while showing lower off-target kinase binding. Unfortunately, MT-802 could not progress further in the *in vivo* drug development process due to unfavorable pharmacokinetic properties.^104^ Another notable development in this field was the synthesis of the PROTAC SJF-620 by Jaime Figueroa’s team.^104^ They used VHL and CRBN ligands while keeping the length of the BTK ligand and linker unchanged. SJF-620 showed promise as an effective strategy for treating C481s mutant chronic lymphocytic leukemia (CLL).^104^ Furthermore, Sun’s research team introduced a new PROTAC strategy to degrade Ibrutinib-resistant BTK specifically.^105^ With high efficiency and specificity, this approach successfully overcame the acquired resistance that resulted from the BTK mutation C481s.^105^

In 2022, Jingyu Zhang et al. conducted a study to use model molecule validation and dimensionality reduction analysis (Principal component analysis (PCA) and discriminant analysis (DA) to find BTK-PROTACs (B1 and B2)) led to improved oral bioavailability and degradation activity and selectivity.^106^ The optimized compounds underwent testing in MDCK cell models to evaluate their permeability, resulting in the discovery of compound C13. Notably, C13 demonstrated enhanced oral bioavailability and remarkable BTK degradation activity.^106^ This resulted in a significant reduction in BTK protein levels and suppression of tumor growth in hematological cancer cells. As a result, C13 exhibits considerable potential as a novel orally bioavailable BTK-PROTAC for lymphoma treatment.^106^

#### 4.1.4 SHP-2 (src Homology 2-Containing Protein Tyrosine Phosphatase 2) PROTACs

A critical role in various signaling pathways, such as RAS-ERK, JAK-STAT, PI3K-AKT, NF-κB, and mTOR, is played by SHP-2, which is a tyrosine phosphatase in the cytoplasm.^107^ Cellular growth, differentiation, and survival depend on these pathways.^107^ As a potential therapeutic approach for treating human cancers and diseases that are linked to the abnormal regulation of these pathways, SHP-2 has attracted significant interest as a target.^108^ In a recent study conducted by Wang et al, potent small-molecule SHP2 degraders were discovered using the PROTAC approach (VHL – E3 ligase).^109^ Among these degraders, SHP2-D26 exhibited remarkable efficacy in reducing the level of SHP2 proteins by more than 95% in cancer cells. It obtained DC_50_ values of 6.0 and 2.6 nm in esophageal cancer (KYSE-520) and acute myeloid leukemia (MV-4–11) cells, respectively.^109^ Compared to the potent SHP2 inhibitor SHP099, SHP2-D26 demonstrated over 30 times greater effectiveness in inhibiting ERK (extracellular signal-regulated kinase) phosphorylation and suppressing growth in this specific cancer cell. Inducing SHP2 degradation is a promising therapeutic strategy for cancers and other human diseases, as these findings show.^109^

In a recent study, Miao et al. discovered a novel PROTAC (P9) designed to target the allosteric site of SHP2.^110^ P9 efficiently degrades SHP2, with a half-maximal degradation concentration (DC_50_) of 35.2 ± 1.5 nM. Notably, P9 exhibits improved antitumor activity across various cancer cell lines compared to its parent allosteric inhibitor. Furthermore, when administered, P9 leads to nearly complete tumor regression in a xenograft mouse model.^110^ This remarkable effect is attributed to robust SHP2 depletion and suppression of phospho-ERK1/2 within the tumor microenvironment.^110^

Importantly, prior to P9, several Cereblon (CRBN)-based SHP2 PROTACs, including ZB-S-29 (DC_50_ = 6.02 nM),^111^ SP4,^112^ and R1–5C,^113^ were investigated. However, these earlier compounds did not demonstrate significant *in vivo* efficacy. In contrast, P9 represents a promising advancement as an effective SHP2 PROTAC with demonstrated *in vivo* activity.^110^

#### 4.1.5 BET (Bromodomain and Extra-terminal Domain) PROTACs

BET proteins comprise two tandem bromodomains and an extra-terminal domain, allowing them to interact with acetylated histones during cell differentiation and proliferation.^114^ These interactions are essential in regulating genetic transcription and affect both latent viral infections and cancer development.^115^ To investigate the modification of BET bromodomain inhibitors, Zengerle et al. utilized different exit vectors and polyethylene glycol (PEG) linkers to VHL ligand VH032.^116^ Their study revealed varying effectiveness among the resulting PROTACs, with triazolodiazepine PROTACs demonstrating higher potency as degraders than tetrahydroquinoline compounds. Furthermore, the length of the linker significantly influenced the BET-degrading and antiproliferative activities.^116^ This research highlights the significance of conjugation in PROTAC development and provides insights into the structure–activity relationships of bivalent degraders.^116^ In a separate study by Qin et al, they discovered QCA570, the most potent and effective BET degrader reported thus far. QCA570 effectively induced BET degradation and inhibited cell growth at low picomolar concentrations in leukemia cells. The IC_50_ values of QCA570 in inhibiting cell growth were 8.3 pM, 62 pM, and 32 pM in MV-4–11, MOLM-13, and RS4–11 blood cells, respectively. Furthermore, when administered to mice carrying leukemia, QCA570 completely eradicated the tumor with long-lasting effects, without causing significant adverse effects even at appropriate intervals.^117^

There have been several reported BET degraders, including ARV-771,^118^ ARV-825,^119^ and BETd-260.^120^ Although these degraders potentially induce BET protein degradation and are more effective than their corresponding BET inhibitors in inhibiting cancer cell growth and inducing apoptosis, even a single atom alteration in PROTAC design can significantly impact the chemical properties and biological activities.^121^ Recently, Ding et al. reported that compound 8b exhibited excellent antiproliferative activity against MM.1S (IC_50_ = 27 nM) and MV-4–11 (IC_50_ = 3 nM) cell lines.^121^ Compound 8b significantly induced the degradation of BRD4 protein and effectively blocked the activation of MRC5 (lung fibroblast) cells. This preliminary evidence suggests that the BRD4 degrader based on the PROTAC concept holds great potential for treating pulmonary fibrosis.^121^ The role of PROTAC in the immunotherapeutic modulation of BET is discussed in Section 3.

#### 4.1.6 HDACs (Histone Deacetylases) PROTACs

HDACs are crucial targets for cancer treatment, but developing drugs specifically targeting individual HDAC isozymes is difficult due to the preserved catalytic domain.^122^ As stated by Smalley et al, Von Hippel-Lindau (VHL), E3-ligase PROTACs were optimized to target HDAC1 and HDAC3 in colorectal carcinoma cells (HCT116).^123^ By modifying the linker length and VHL ligand, PROTAC molecules 7, 9, and 22 were identified, effectively targeting and degrading HDAC1 or HDAC3 with micromolar DC_50_ values. Compound 7 exhibited DC_50_ values of 0.91 ± 0.02 μM for HDAC1 and 0.64 ± 0.03 μM for HDAC3. Compound 9 displayed comparable DC_50_ values measuring at 0.55 ± 0.18 μM (HDAC1) and 0.53 ± 0.13 μM (HDAC3). Compound 22 demonstrated a notable inhibitory effect on HDAC3, with a DC_50_ value of 0.44 ± 0.03 μM. By changing the position of the VHL ligand attachment to the linker, the researchers were able to overcome the “hook effect” for HDAC3. In HCT116 cells, the HDAC1/2 degraders with greater potential led to an increase in all differentially expressed genes and an improvement in apoptosis. The study revealed that the use of PROTACs to degrade HDAC1/2 was associated with an increase in global gene expression and apoptosis, suggesting the possibility of developing more effective HDAC therapeutics with fewer side effects.^123^

Specifically, PROTACs have shown promise in targeting HDACs, which play a critical function in inflammatory diseases such as asthma and chronic obstructive pulmonary disease. The adverse effects of many HDAC inhibitors have hampered their therapeutic potential for both cancerous and noncancerous conditions. PROTACs offer a new approach by enhancing HDACs binding and reducing side effects. For instance, PROTACs targeting HDAC 1/3 (HDAC degrader) have been developed by connecting hydroxamic acid and benzamide with lenalidomide, pomalidomide, and cc 220 using various lengths and different types of linkers. Studies have demonstrated that the length and type of linkers in HDAC 1/3 degraders impact their function and antiproliferative activities in cells.^124^ Similarly, PROTACs targeting HDAC 1/2/3 have also been developed, with PROTAC 2 showing the highest activity as a degrader. PROTAC 2 consists of benzamide HDAC inhibitors, an alkaline linker molecule, and a VHL ligand.^125^

Numerous PROTAC HDAC degraders have been reported, showing great potential for use in tumor immunotherapy.^126^ Specifically, VHL E3 ligase-recruiting PROTACs, such as JPS004, exhibit degradation of HDAC1/2 and HDAC3. Minor modifications to the VHL E3 ligand can selectively target HDAC3 over HDAC1/2, as demonstrated by JPS036.^127^ Additionally, other selective HDAC3 degraders utilize VHL and CRBN-recruiting E3 ligase ligands, including PROTACs HD-TAC7^128^ and XZ9002.^129^

#### 4.1.7 Bcl-2 (B-Cell Lymphoma 2) PROTACs

Bcl-2 is an apoptosis-resistant molecule associated with cancer, which plays an important role in regulating apoptosis.^130^ Bcl-2 PROTACs, utilizing an E3 ligase, selectively induce the Bcl-2 degradation. The using the PROTACs in living cells has demonstrated reversible depletion, offering a novel approach to examine the Bcl-2 and Mcl-1 (myeloid cell leukemia-1) dynamic functions in apoptosis. TPD with PROTACs represents a potential solution to overcome drug toxicity on target. Until now, however, only two PROTAC compounds (C3 and C5) have been reported to selectively degrade Bcl-2/Mcl-1 in apoptosis network.^131^

DT2216, a PROTAC, is designed by linking ABT263 (a dual inhibitor of BCL-xL and BCL-2) with a VHL E3 ligase binding ligand.^132^ Unlike ABT263, DT2216 exhibits reduced platelet toxicity because VHL is minimally expressed in platelets, limited BCL-xL.^132^ Surprisingly, DT2216 forms a ternary complex with both BCL-xL and BCL-2 *in vitro*, yet it effectively degrades only BCL-xL, not BCL-2, in cells. Subsequently, the development of 753b, the first BCL-xL/BCL-2 dual degrader, improve on DT2216 in potency.^133^ However, the molecular mechanisms underlying the specificity of these PROTACs remain unknown due to the absence of structural studies. Recently, Nayak et al. elucidated crystal structures of VHL/753b/BCL-xL and BCL-2, shedding light on their interactions.^134^ DT2216 is already in clinical trials (T cell lymphomas) as the unique PROTAC degrader targeting BCL-xL.

#### 4.1.8 NAFLD (Nonalcoholic Fatty Liver Disease) Immune Target PROTACs

NAFLD is a condition characterized by fat accumulation in the liver without other underlying causes. When the fat build-up exceeds 5% of hepatocytes, it is considered pathological.^135^ NAFLD encompasses two conditions, namely nonalcoholic fatty liver (NAFL) and nonalcoholic steatohepatitis (NASH), with NASH involving inflammation, liver cell damage, and fat accumulation.^135^ In a study conducted by Yang Yang et al, liver-tropic senolytic activity reported with compound 753b (BCL-xL PROTAC).^136^ This compound is a liver-targeted BCL-xL PROTAC with potent properties. The researchers observed that 753b can uniquely eliminate senescent hepatocytes in aged mice and NASH-driven hepatocellular carcinoma (HCC) model (STAM mice), despite its ability to effectively kill various senescent cells *in vitro*. This selectivity is attributed to its liver enrichment after intraperitoneal injection. Furthermore, treatment with 753b was initiated to reduce the incidence of NASH, liver fibrosis, and HCC in STAM mice. These results indicate that 753b is promising as a potential therapeutic option for NAFLD and NASH-related HCCs.^136^ Furthermore, NASH has shown that it limits antitumor surveillance of HCC treated with immunotherapy by promoting the exhaustion of CD8+ PD1+ T cells in the liver, suggesting a potential therapeutic strategy to improve the immune therapy response in this environment.^137^ NASH-related HCC has a unique immune microenvironment that influences its pathogenesis and response to therapy.^138^

Fatty liver disease (FLD) is caused by the accumulation of triglycerides (TGs) in the liver and leads to inflammation, fibrosis, and cirrhosis. The PNPLA3 (patatin-like phospholipase domain-containing protein 3) gene variant I148 M has been associated with FLD, but the underlying mechanism is not fully understood. Previous studies have shown that overexpression of wild-type proteins (PNPLA3) in mice does not cause steatosis. However, the expression of mutated forms of PNPLA3 (I148 M or S47A) in mice who consumed a sucrose diet leads to increased PNPLA3 and TGs on hepatic lipid droplets. To investigate whether PNPLA3 protein accumulation is the cause of steatosis, researchers developed a synthetic isoform of PNPLA3 that dissociates protein accumulation from loss of enzymatic activity. By expressing a form of PNPLA3 that is resistant to ubiquitylation in mice, the study showed that PNPLA3 accumulated on lipid droplets in the liver and caused FLD to develop. In addition, reducing PNPLA3 levels through the knockdown of shRNA or degradation by PROTAC reduced the liver TG content in mice overexpressing PNPLA3(148M). In summary, the research findings demonstrate that the buildup of PNPLA3 on lipid droplets within the liver is responsible for developing steatosis linked to PNPLA3(148M) varian.^139^ Another strategy to improve NAFLD is to target the Keap1/Nrf2 pathway, which regulates the antioxidant response and lipid metabolism in the liver. Keap1, an E3 ubiquitin ligase, targets Nrf2 degradation, a transcription factor crucial for detoxification and lipid balance gene expression. A recent study discovered a series of small molecules that bind to Keap1, and disrupt its interaction with Nrf2, leading to increased Nrf2 levels and activity.^140^ The study showed that PROTAC I-d improved NAFLD in mice fed a high-fat diet by reducing hepatic TG content, inflammation, innate immune signaling, and oxidative stress.^140^ These findings suggest that promoting the degradation of both Keap1 and PNPLA3 could be an effective therapeutic approach for NAFLD.^139,140^

Fengqin Wang and colleagues developed a chimeric Keap1 peptide (KKP1) using PROTAC technology to induce the degradation of Keap1 protein through the UPS pathway. This guides to the release of Nrf2 (nuclear factor erythroid 2-related factor 2) and initiation of the Nrf2 and antioxidant response element pathway. Consequently, the expression of downstream antioxidant factors, such as heme oxygenase-1 and glutamate-cysteine ligase catalytic subunit, is promoted, while the nuclear factor-kappaB inflammatory signal pathway, inflammatory factors (tumor necrosis factor-α and interleukin-1β), and fibrosis biomarker gene activation are inhibited. The KKP1 peptide effectively penetrates the HSC-T6 cells (rat hepatic stellate cells), suggesting its potential as a therapeutic approach for diseases related to oxidative stress.^141^

In a recent study, Park et al. developed SD2267, a proteolysis-targeting chimera (PROTAC), which induces CRBN-mediated proteasomal degradation of KEAP1 in hepatocytes.^142^ SD2267 translocates NRF2 into the nucleus and increases the transcription of its target genes, including HMOX1, NQO1, GCLC, and GCLM. Remarkably, this is the first instance where PROTACs have demonstrated an *in vivo* antioxidative effect.^142^

#### 4.1.9 Cyclooxygenase 1/2 (COX-1/2) PROTACs

Prostanoids are synthesized by prostaglandin G/H synthase, also known as cyclooxygenase (COX), acting on arachidonic acid.^143^ COX, an evolutionarily conserved bifunctional enzyme, exists in two distinct isoforms: COX-1 and COX-2.^143^ While COX-1 is constitutively expressed in most cells and serves as the primary source of prostanoids for essential functions like gastric epithelial cytoprotection and hemostasis, COX-2 is often upregulated in various disease conditions.^143^ It is considered a potential therapeutic target for anti-inflammatory treatments but may also play a role in colon cancer and Alzheimer’s disease.^144^ COX-2 inhibitors have demonstrated anticancer properties across different cancer types.^145,146^ Researchers have developed a smart nano-PROTAC (SPN_COX_) with phototherapeutic capabilities to degrade COX-1/2, thereby remodeling the tumor microenvironment for cancer immunotherapy.^147^ Activation of the COX-1/2 PROTAC by cathepsin B overexpression leads to COX-1/2 degradation, resulting in reduced prostaglandin E2 levels and enhanced anticancer immune responses.^147^ In summary, combining the COX-1/2 PROTAC with phototherapy reactivates the tumor microenvironment and improves the efficacy of immunotherapy.

#### 4.1.10 STAT3 and IRAK4 PROTACs

Signal transducer and activator of transcription 3 (STAT3) is continuously activated or overexpressed in a variety of malignant cells.^148^ It can be activated by cytokines and growth factors. STAT3 activation inhibits antitumor immune responses.^149^ Shaomeng Wang research team designed and synthesized a series of potential STAT3 degraders the development of a small-molecule PROTAC targeting STAT3.^150^ Known as SD-36, this PROTAC potently and selectively degraded STAT3 protein lymphoma and leukemia cells, while also mediating complete tumor regression in mouse tumor models.^151^ The warhead for SD-36 was the small molecule SI-109, a STAT3 SH2 domain inhibitor reported to bind to STAT3 with high affinity. To generate the SD-36 PROTAC, SI-109 was attached via a six-carbon linker to a ligand analog of lenalidomide, which acts to recruit the CRBN E3 ligase.^150,151^

Furthermore, a recent study by Lin et al. discovered that PJ-001 degrader improves atopic dermatitis (AD) inflammation in mice by inhibiting the JAK2/STAT3 pathway and repairing the skin barrier.^152^ These findings provide direct evidence that PJ-001 effectively mitigates inflammatory infiltration, thus improving skin itching and epidermal keratinization. PJ-001 can reduce the inflammatory response in a mouse model of AD by inhibiting the activation of inflammatory pathways. In particular, KT-474 is a potential first-in-class Interleukin-1 Receptor-Associated Kinase 4 (IRAK4) degrader that is being developed for the treatment of TLR/IL-1R-driven immune inflammatory diseases (Table 3), Table such as AD.^68^ Nunes et al. discovered IRAK4 degradation molecule, compound 9 to the inhibition of cytokines of peripheral blood mononuclear cells.^153^ IRAK4 degrader-5 characterized as a pharmacological tool to examine the enzymatic and scaffolding functions of IRAK4 in activated B-cell-like diffuse large B cell lymphoma (ABC DLBCL).^154^ The utilization of PROTAC technology holds promise as a novel approach in drug development for the treatment of immune-inflammatory diseases.

#### 4.1.11 Other Targets H-PGDS, NAMPT, RIPK2, and SIRT2 PROTACs

Other promising targets for PROTAC-mediated degradation include H-PGDS, NAMPT, RIPK2, and SIRT2. These molecules play crucial roles in various disease processes, and their targeted degradation using PROTAC technology holds therapeutic potential for a variety of conditions.

H-PGDS, a target for diseases such as allergies and Duchenne muscular dystrophy, currently lacks approved drugs. PROTAC(H-PGDS)-1, a molecule designed to degrade H-PGDS through the ubiquitin proteasome system.^155^ PROTAC(H-PGDS)-1 effectively reduces the production of the H-PGDS protein and prostaglandin D2 (PGD2), with sustained effects even after drug removal.^155^ This suggests promise for PROTAC(H-PGDS)-1 in both research and future therapies. Building on this success, the researchers used computer simulations to develop an even more potent degrader, PROTAC(H-PGDS)-7 (DC_50_ = 17.3 pM).^156^ This new molecule not only showed strong suppression of PGD2, but also better inhibition of inflammatory cytokines in a muscular dystrophy model compared to a traditional H-PGDS inhibitor.^156^

NAMPT, a critical player in cancer metabolism and inflammation, is emerging as a promising therapeutic target. PROTAC A7 effectively degrades both intracellular and extracellular NAMPT, leading to superior antitumor activity compared to traditional inhibitors.^77^ This success has encouraged the development of a robust pipeline of NAMPT degraders.^157^ Compounds 630120/630121 exhibit activity in various tumor models,^158^ while B3 shows exceptional degradation (DC_50_ < 0.17 nM, *D*_max_ > 90%) and antiproliferative effects (IC_50_ = 1.5 nM).^159^ In particular, B4, a fluorescent PROTAC, allows for degradation visualization.^160^ Research has further expanded into next-generation strategies such as semiconducting polymer NanoPROTACs, which not only degrade NAMPT but also suppress myeloid-derived suppressive cells and promote antitumor immunity.^161^ The drugtamer-PROTAC conjugation strategy holds promise for the targeted delivery of PROTACs with synergistic drugs for NAMPT-based therapy.^162^ Finally, the *in vivo* active LYP-8 demonstrates promise as a potential novel cancer therapy.^163^ Together, these advances highlight the immense potential of PROTAC technology for effective and targeted NAMPT degradation in cancer treatment.

RIPK2, a key mediator of innate immunity, emerges as a promising target for PROTAC-mediated degradation. Early studies like PROTAC_RIPK2 demonstrated dose-dependent and specific degradation in immune cells.^164^ Building on this success, GSK’s PROTAC 6 achieved concentration and time-dependent RIPK2 reduction in human immune cells, with a unique pharmacologic advantage: repeated submaximal doses caused progressive RIPK2 degradation without drug accumulation.^165^ Furthermore, their PROTAC compound 20 offered high potency, selectivity, solubility, and favorable drug metabolism properties.^166^ Recent advancements include Chan et al. development of an antibody-PROTAC conjugate that selectively degrades RIPK2 in HER2+ cancer cells. This approach complements existing antibody drug conjugates and provides a strategy for PROTACs with suboptimal properties or for targeted delivery.^167^ These findings highlight the promise of PROTAC technology for targeted RIPK2 degradation in various therapeutic areas.

SIRT2, implicated in cancer and neurodegeneration, is a promising target for targeted degradation using PROTACs. Early strategies included SirReal-derived PROTACs and structure-based development of SIRT2 degrading molecules.^168,169^ Recent work by Hong et al. introduced TM-P4-Thal, a PROTAC that degrades SIRT2 and inhibits its enzymatic activities,^170^ mimicking SIRT2 knockout effects in mice.^171^ Furthermore, activity-based probes (ABPs) were developed for SIRT2 visualization and capture.^172^ Notably, cell-permeable probe 3A served as the foundation for a SIRT2 PROTAC (PRO-SIRT2) demonstrating efficient, concentration-dependent SIRT2 degradation via the ubiquitin-proteasome system.^172^ These findings solidify the potential of PROTAC technology for targeted SIRT2 degradation in therapeutic areas. Table 3 provides a comprehensive summary of the various types of PROTACs in clinical trials and immunotherapy-related targets.

## 5 AI-Powered PROTAC Development and DMPK

Artificial intelligence (AI)-driven PROTAC development involves utilizing machine learning and deep learning algorithms to optimize molecule structure and predict interactions with target proteins and E3 ubiquitin ligase.^173^ This approach accelerates the development of efficient and specific PROTACs. Computational modeling, including protein–protein docking and molecular dynamics simulations, aids in rational design. PROTACs face a few challenges, such as optimizing the linker length and composition, selecting the appropriate E3 ligase, and avoiding unwanted degradation of bystander proteins.

**Figure 7:**
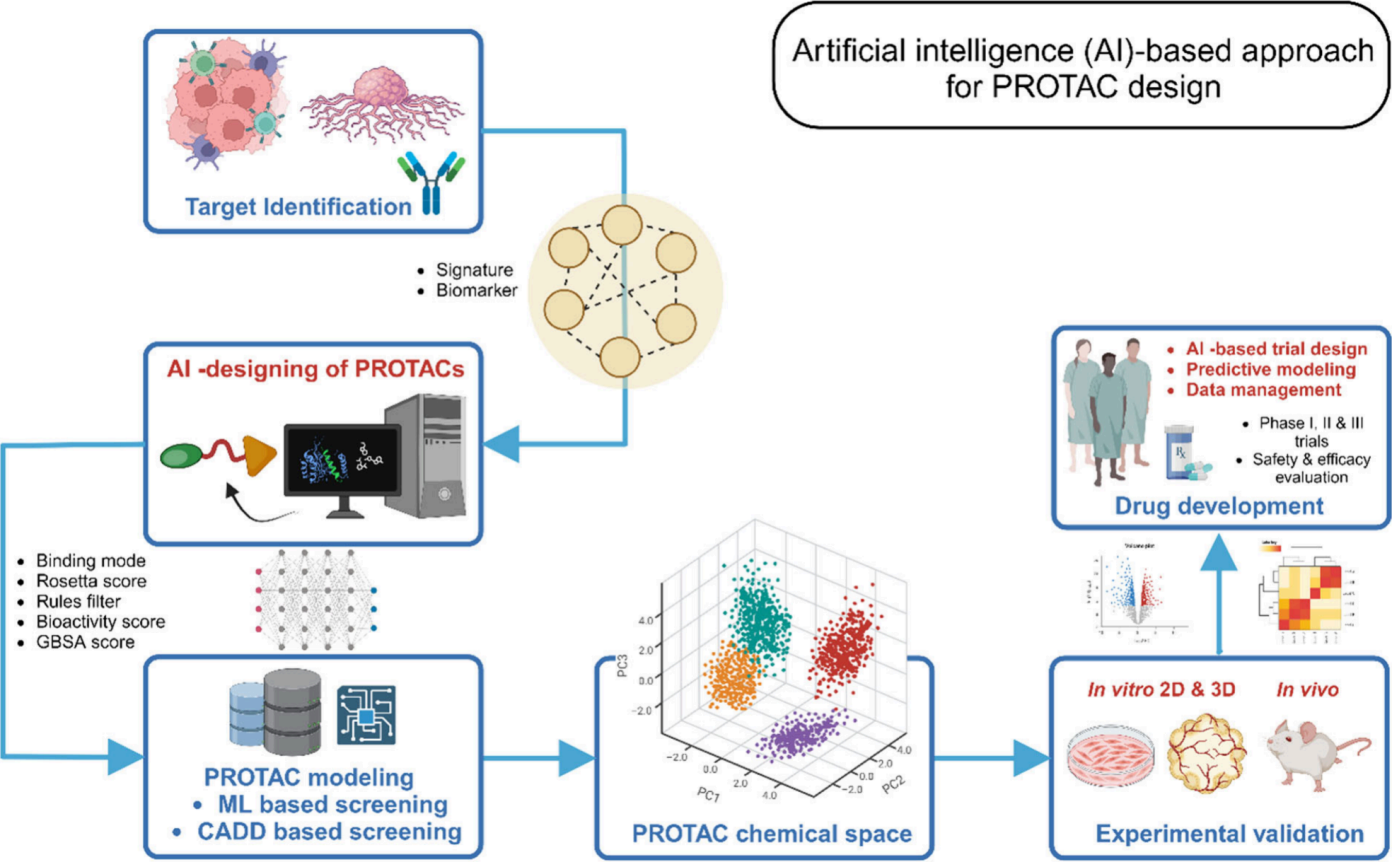
Flowchart showing different steps in PROTAC development using AI.

Computational modeling is playing an increasingly important role in TPD research.^51^ Various strategies and pipelines, such as PRosettaC and RosettaDock, utilize docking algorithms and molecular dynamics (MD)-based protocols to predict ternary complex formation and guide PROTAC design.^51^ Combining FRODOCK and RosettaDock has shown success in reproducing native conformations,^174^ while machine learning techniques like Bayesian Optimization and deep learning enhance complex modeling and degradation efficiency predictions.^175^ Computational models have also been developed to predict ubiquitination processes by considering E3 complex dynamics and structural patterns.^176^ In the context of targeted cancer therapy, a computational framework called PROTAC-RL has been developed to design PROTACs with optimal properties.^177^ This framework combines generative modeling, machine learning, and physics-driven learning, resulting in experimentally validated PROTACs that exhibit effective anticancer efficacy and pharmacokinetic properties. The framework achieved a success rate of 50% and a turnaround time of 49 days, demonstrating its potential for accelerating the discovery of promising drug candidates when combined with artificial intelligence-driven computational strategies and experimental validation.^177,178^Figure 7 demonstrates a flowchart showing different steps in PROTAC using AI.

Nowak et al. showed an example of computational-powered PROTAC development by using the Rosetta dock to perform protein–protein docking and design a degrader that can selectively target BRD4 among the closely related BET bromodomains of BRD2/3/^4178^. They synthesized a potent and specific BRD4 degrader that proved the effectiveness and benefits of using AI to design PROTACs for immunotherapy. Similarly, the HADDOCK server is used for molecular modeling of the ternary complex of SIRT2 and HaloTag 7 (HT7) by PROTAC compound 12.^169^

PROTAC design is challenging due to complex structure–activity relationships. A recent study introduced DeepPROTACs, a deep learning model leveraging PROTAC-DB data (DC_50_ and *D*_max_ values) to predict molecule potency.^179^ DeepPROTACs utilizes graph convolutional networks to analyze ligand-protein interactions and achieves high accuracy (77.95%) in predicting successful PROTAC designs.^179^

The docking of the ternary complex method predicts the structure of the ternary complex using *in silico* approaches, such as docking, molecular dynamics, and machine learning. Molecular dynamics simulations were applied to design the macrocyclic PROTAC MZ1.^180^ A common tool for modeling PROTAC-mediated ternary complexes is ProsettaC, which integrates global docking with PatchDock under PROTAC-derived distance constraints and local docking with RosettaDock, and then models the PROTAC into the ternary complex. This can facilitate the design of new PROTACs for various targets.^181^ Recently, the ProsettaC web server was employed to design the KH-103 PROTAC molecule that targets glucocorticoid receptors for stress-related neuropsychiatric disorders *in vivo*.^182^

**Figure 8:**
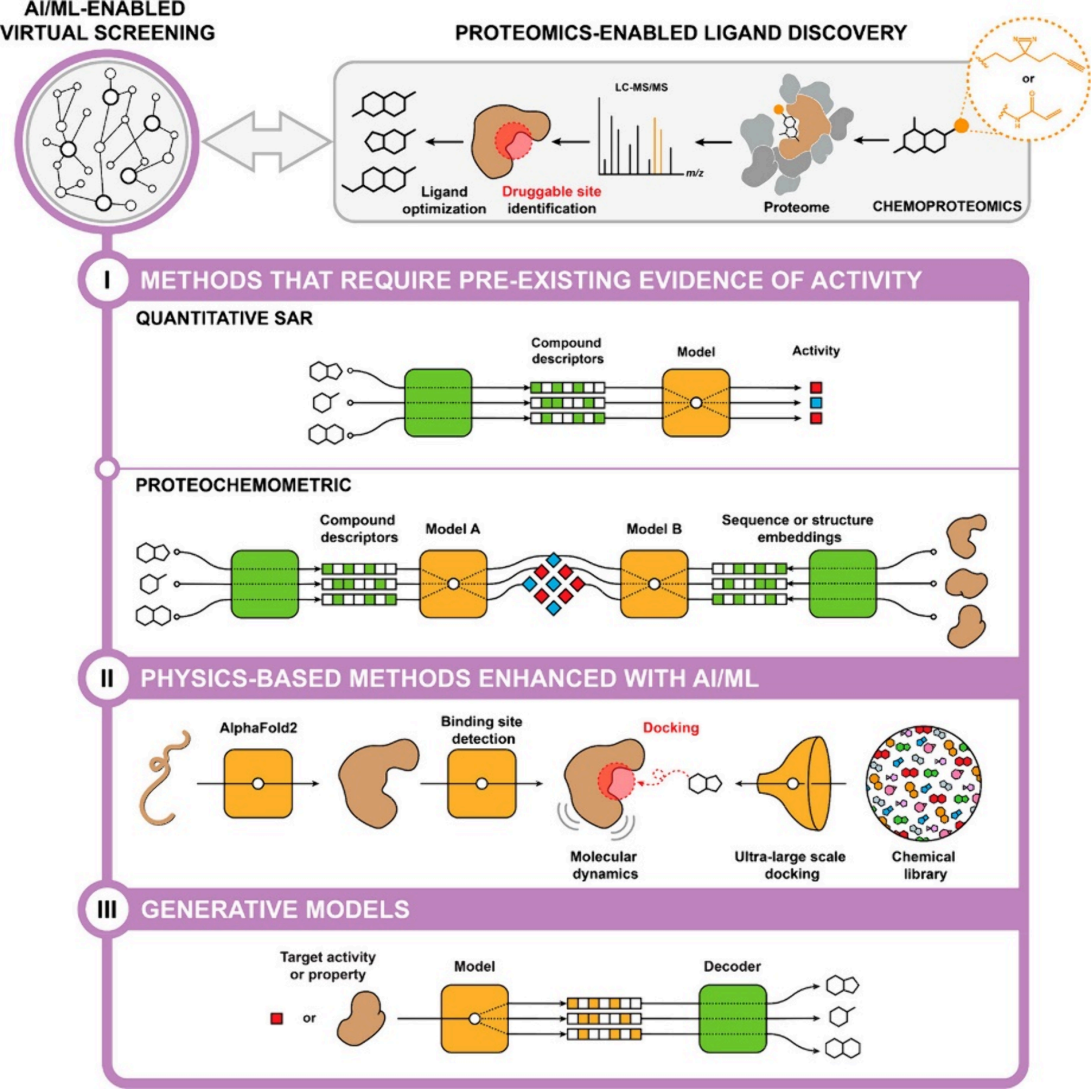
Advancing ligand discovery with proteomics and computational approaches is a field that encompasses various methodologies, including AI/ML-based methods.^50^ These methods can be categorized into three distinct groups (I–III) based on the type of input information they utilize. Adapted with permission from ref (50) under CCBY 4.0 License.

AI speeds up PROTAC development by predicting success and guiding design of potent, selective molecules. It tackles challenges in protein degradation, paving the way for better drugs and improved therapies. Conventional drug discovery is a slow and expensive process, often taking years and costing billions with a high chance of failure. AI-powered PROTAC design offers a promising alternative, potentially accelerating drug development, reducing costs, and increasing success rates.^177^Figure 8 depicts how ligand discovery for PROTACs can be done with AI/ML.^50^

Crystal structures of PROTAC-induced ternary complexes (18 ternary complex structures) reveal flexibility despite diverse protein targets. This inherent mobility within E3 ligase assemblies allows for widespread protein ubiquitylation, suggesting that rigid structures are not essential for degradation. Mathematical modeling confirms this, showing that protein dynamics does not directly correlate with degradation efficiency. Interestingly, salt bridges within these complexes contribute to stability and potentially enhance degradation. These findings suggest that the PROTAC design should prioritize presenting lysine residues near the E2 enzyme, while managing, not eliminating, dynamic interactions for optimal protein degradation. These findings highlight the importance of studying a wider range of PROTAC-target interactions.^183^

However, the full potential of computational approaches to exploit these findings is hindered by the limited number of structurally/biophysically characterized ternary complexes, especially those beyond CRBN/VHL and BET proteins. High-quality structural and biophysical data are crucial for developing computational models to design new PROTACs. Studying ternary complex formation and degradation (*in vitro* and in cells) helps refine these models. Initiatives like PROTAC-DB improve data sharing, accelerating breakthroughs in both experimental and computational PROTAC development.^51^

Degraders, a promising therapeutic strategy, pose unique challenges due to their large size and physicochemical properties. These features, often exceeding the “rule of five” for drugs, result in poor solubility, permeability, and potentially high clearance rates.^184^ Key DMPK barriers: 1) Solubility: Limited aqueous solubility hinders oral absorption. Solubility optimization is critical for successful oral degraders. 2) Permeability: Degraders generally have low permeability due to their size and flexibility. While desirable, permeability improvements are secondary to solubility. 3) Metabolism: Degraders can be metabolized by enzymes like CYP450 and UGT, impacting their exposure. Strategies to improve metabolic stability are necessary.^185^

**Figure 9:**
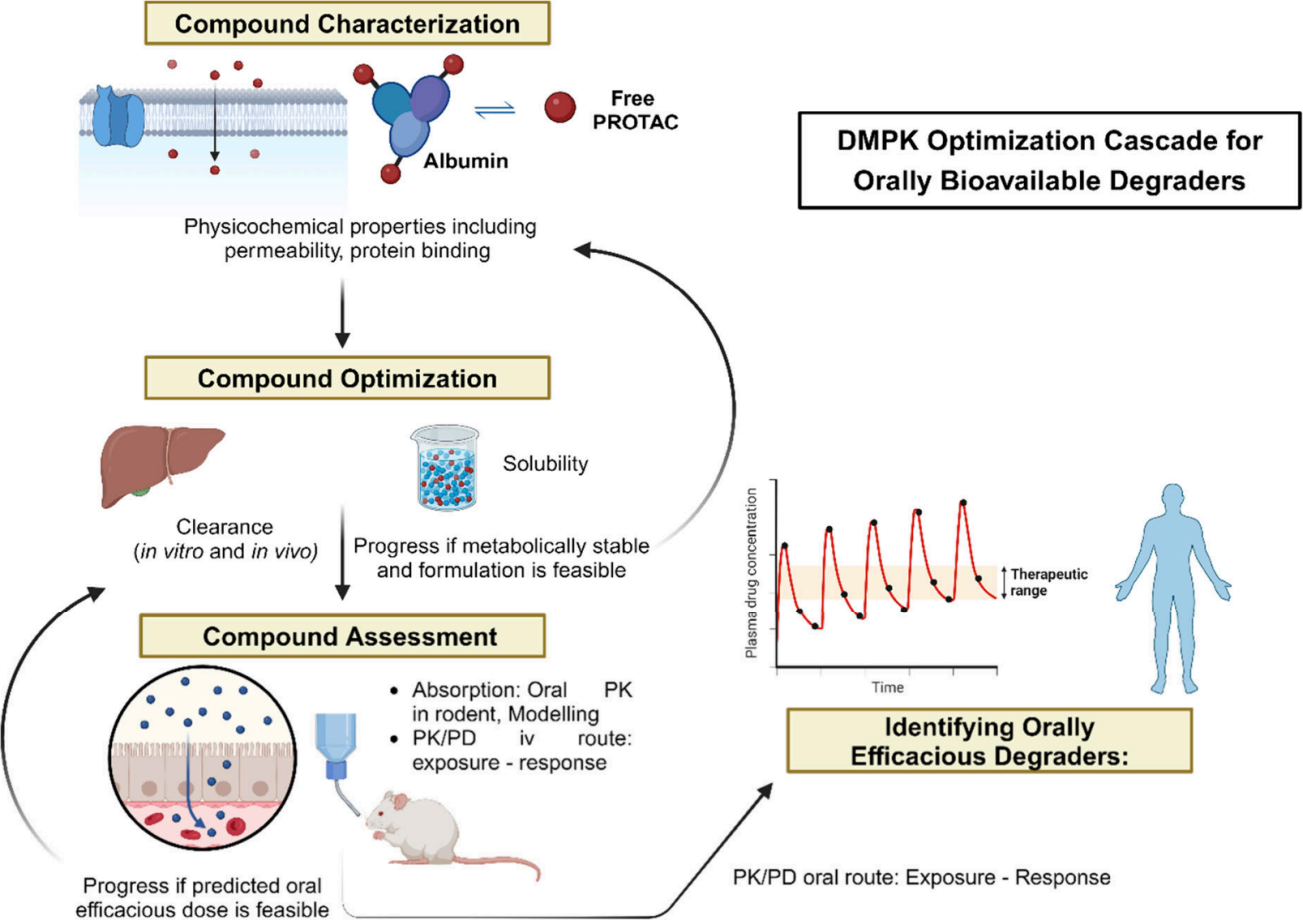
DMPK Optimization Cascade for Identifying Orally Bioavailable Degraders. This schematic depicts the key steps involved in the preclinical DMPK (drug metabolism and pharmacokinetics) optimization cascade used to identify PROTAC degraders suitable for oral administration. The cascade focuses on optimizing various properties of the degrader molecule to ensure successful oral delivery: 1. Compound Characterization: Initial evaluation of the degrader molecule’s physicochemical properties, including permeability and protein binding. 2. Compound Optimization: Iterative cycles of testing and refinement to improve the degrader’s metabolic stability and solubility, both crucial factors for oral bioavailability. 3. Compound Assessment: Evaluation of the optimized degrader’s oral pharmacokinetics (PK) in rodent models, often accompanied by PK modeling to understand absorption mechanisms. 4. Identification of Orally Efficacious Degraders: Selection of degrader candidates demonstrating both potency and good oral bioavailability for further PK/PD studies. Investigation of the relationship between degrader exposure and its pharmacodynamic (PD) effects, such as tumor growth inhibition. By navigating this cascade, researchers can identify promising PROTAC degraders with the potential to be developed into orally administered therapeutics.^184^

Studies have shown cellular assays to be more reliable than PAMPA for permeability assessment of degraders.^184^ Thermodynamic solubility experiments offer more accurate data than traditional methods. Ultracentrifugation is a suitable approach for measuring protein binding. A focus on solubility optimization through DMPK refinement is essential for achieving orally bioavailable degraders. Preclinical PK–PD studies can then elucidate the relationship between drug exposure and target inhibition, guiding translation to human trials. Further research is needed to fully understand the impact of degrader properties on target engagement and tumor growth inhibition. This knowledge is crucial for designing future generations of orally bioavailable degrader therapeutics. Figure 9 depicts general DMPK optimization strategy for discovering orally bioavailable degraders.^184^

## 6 Key Challenges and Opportunities for PROTAC

The field of targeted protein degradation has seen rapid growth in the past decade, with numerous degradation molecules entering clinical trials for various diseases (Table 3). However, there are still many hurdles to overcome and many possibilities to pursue in this emerging area of drug discovery. In the next section, we will discuss some of the most promising opportunities and challenges that lie ahead for TPD drug development.

### 6.1 Designing PROTAC

Designing PROTAC molecules and other biologics for TPD is challenging. Unlike traditional small molecules, PROTACs, unlike antibodies and oligonucleotides, offer good permeability and bioavailability due to their small molecule nature. However, they often violate Lipinski’s “Rule of Five,″ posing challenges for therapeutic development. Additionally, PROTAC design remains largely an empirical process, limited to well-characterized E3 ligases like VHL and CRBN. Modifying PROTACs to achieve drug-like properties and favorable pharmacokinetics/pharmacodynamics is often time-consuming and laborious compared to smaller molecules.^186^

The traditional limitations of drug design are being challenged by “beyond-the-rule-of-five” (bRo5) drugs, including PROTACs, which demonstrate oral bioavailability despite violating Lipinski’s Rule.^187^ Assessing these bRo5 drugs requires a nuanced approach. The AbbVie multiparametric score (AB-MPS) goes beyond traditional metrics by incorporating factors like membrane permeability (LogD), aromatic rings, and rotatable bonds.^187,188^ The “bRo5 and PROTACs in modern drug discovery” explores deeper into the concept of optimizing physicochemical properties for PROTACs. Recently, it proposed the use of the experimental polarity surface area (EPSA) alongside topological polar surface area (TPSA) to create a more informative metric: the EPSA-to-TPSA ratio (ETR).^188^ This ratio helps assess a compound’s ability to reduce its effective polarity due to external factors like molecular shielding or dynamic interactions, potentially a form of “chameleonicity.″

AB-MPS and EPSA, while valuable for absorption trends and physicochemical understanding, are limited by retrospective analysis.^188^ Comprehensive absorption assessment requires additional tools (cellular systems, predictive models) to evaluate factors like active transport and gut metabolism. Nevertheless, integrating a more nuanced view of size, polarity, flexibility, and “chameleonicity” (via ETR) with AB-MPS and EPSA can streamline PROTAC development for improved absorption. This empowers discovery teams for earlier informed decisions, accelerating the path from PROTAC concept to therapeutic agent.^188^

### 6.2 Knowledge Gap of E3 Ligases

Although there is a vast majority of E3 ligase availability, currently we are using approximately 12 E3 ligases, including CRBN, VHL, IAPs, MDM2, AhR, DCAF15, DCAF16, DCAF11, RNF114, KEAP1, FEM1B, and RNF4.^34^ Identifying specific ligands for E3 ligases can be challenging due to the lack of known ligands for many of these proteins.^189^ However, the discovery of thalidomide binding to CRBN E3 ligases provides insight into the identification process.^190^ The identification involved a combination of biochemical studies, structural analysis, and functional assays. Researchers used a combination of cocrystal structures and mutational analysis to identify key interactions between thalidomide and CRBN, highlighting the importance of specific residues in ligand binding. These findings not only demonstrated the binding of thalidomide to CRBN but also provided a framework for understanding the molecular basis of ligand recognition by E3 ligases.^189,190^

Before we can fully utilize the ability of these proteins as cancer therapies, there are still several important gaps in our knowledge regarding the >700 E3 ligases found in the human genome.^191^ Recent analyses have identified promising candidates for development as extant (pre-existing) PROTACs by evaluating several E3 ligases. By combining factors like confidence score, ligandability, expression pattern, and protein–protein interactions, researchers were able to pinpoint 76 E3 ligases with high potential for PROTAC development.^191^ While recent studies have identified promising E3 ligases for PROTAC development, significant knowledge gaps remain. One key area is understanding the tissue-specific expression patterns of E3 ligases, which could enable targeted protein degradation in specific tissues.^192^ Additionally, developing more robust methods for identifying E3 ligase ligands is crucial. The direct binding assays stand out as an attractive approach due to their simplicity and effectiveness.^192^ DNA-encoded libraries (DELs) offer a particularly exciting opportunity, as they can rapidly screen vast chemical spaces for potential E3 ligase binders in a single experiment.^192^

### 6.3 Solubility Issues of PROTAC

PROTACs hold immense therapeutic promise for targeting previously undruggable proteins. However, their complex structure, often incorporating hydrophobic and polar domains, presents a major hurdle - poor solubility. This limited solubility hinders bioavailability and impacts pharmacokinetic profiles.

Several factors contribute to this challenge: I) High hydrophobicity: Many PROTACs exhibit poor water solubility, leading to low bioavailability and suboptimal pharmacokinetics. II) Aggregation: Hydrophobic interactions within the molecule can cause aggregation and precipitation, potentially leading to off-target effects. III) Nonspecific binding: Reactive groups or ligand similarities can result in unintended protein interactions and toxicity. Strategies like prodrugs, nanosuspensions, and linker/ligand optimization are being explored to address these issues.^193^ Predicting solubility through methods like general solubility equation (GSE) can also aid development.^194^ Overall, overcoming solubility limitations remains a critical step in realizing the full potential of PROTACs for clinical use.

### 6.4 Nanomaterial-Based PROTACs Delivery System in Immunotherapy

To date, PROTACs still face the challenges of absorption, distribution, metabolism, excretion, and toxicity, which results in low bioavailability, limiting their development and application.^195^ In an early effort, Wang and his team synthesized and developed a nanoplatform for gold nanoparticle-based multiheaded PROTACs targeting anaplastic lymphoma kinase (ALK), using lung adenocarcinoma cell lines (NCI-H2228). The ALK inhibitor ceritinib and E3 ligase ligand pomalidomide were conjugated to the surface of the gold core through thiol-modified polyethylene glycols (PEGs). These acted as a spacer or linker to develop the multiheaded PROTACs (Cer/Pom-PEG@GNPs). The nano-PROTAC synthesized had diameters <200 nm, and cell uptake was confirmed with a transmission electron microscope. Western blotting confirmed the dose-dependent degradation of ALK protein, but a hook effect was observed at high concentrations. Cer/Pom-PEG@GNPs demonstrated cell-specific cytotoxicity effects, although animal studies were notably absent from these investigations.^196^ In this context, gold nanoparticles are designed to create a peptide-based nano-PROTAC. This nano-PROTAC exhibits strong degradation activity against MDMX (also referred to as MDM4). It has been tested in a patient-derived xenograft model of pancreatic cancer and has shown a safe profile.^197^

In the field of cancer-activated optical immunometabolic therapy, PROTAC molecules are explored as intelligent activable semiconducting polymer nano-PROTACs (SPNpro). SPNpro is composed of a semiconducting polymer material core, which is conjugated with PROTAC segments via a cancer-biomarker-cleavable peptide (cathepsin B). In this research, indoleamine 2,3-dioxygenase (IDO) was chosen as the protein of interest due to its suppressive effect on T cells. The IDO-targeting PROTAC peptide (IPP) consists of an IDO targeting unit (a widely used IDO inhibitor, NLG919) and an E3 ubiquitin ligase VHL (the von Hippel Lindau protein)-binding peptide.^43^

Upon systemic administration, SPNpro accumulates in the tumor with the aid of the biomarker cathepsin B (CatB). The tunable properties of these semiconducting polymer nanoparticles are utilized to release singlet oxygen (^1^O_2_) to eliminate tumor cells and induce the release of tumor-associated antigens and immunogenic cell death (ICD) under near-infrared photoactivated irradiation. The released tumor-associated antigens further stimulate dendritic cells (DCs) maturation and promote T-cell activation, enabling an antitumor T-cell immune response.^43^ Simultaneously, the overexpressed tumor CatB cleaves SPNpro and releases PROTAC molecules in situ. The activated form of PROTAC binds to the target protein (IDO) and brings it to the E3 ubiquitin ligase VHL ligand, leading to persistent IDO degradation via the ubiquitin-proteasome system. The degradation of IDO alleviates the overconsumption of the Trp-catabolizing enzyme and accumulation of kynurenine, leading to the reversal of immune suppression. This advanced concept was validated with a mouse model, where SPNpro effectively suppressed tumor progression.^43^ The same team has further expanded this concept to smart Nano-PROTACs that reprogram the tumor microenvironment for cancer immunotherapy, specifically targeting cyclooxygenase 1/2 (SPNcox) and Src homology-2 domain-containing protein tyrosine phosphatase-2 (checkpoint nano-PROTAC-NPRO).^198^ They have managed to enhance antitumor immunity and decrease immune suppression in *in vivo* studies.^147,198^

On the flip side, the nanoformulation of PROTAC molecules is being developed. ARV-825 shows great potential in treating vemurafenib-resistant melanoma that targets BRD4, although it encounters solubility issues. The team led by Ketan Patel has formulated ARV-825 using the PEGylated nanoliposome concept to enhance delivery and penetration into the 3D spheroid model.^199,200^

Zhang and colleagues engineered a nano-PROTAC known as CREATE, designed specifically to degrade BRD4. The construction of these nanoparticles involved the use of a pH/glutathione (GSH)-responsive polymer, specifically disulfide bond-linked poly(lactic-*co*-glycolicacid), or PLGA–S–S-PLGA (DS-PLGA). This was used to load dBET6, a BRD4 degrader with limited bioavailability. This was then combined with CRV-engineered Lewis lung carcinoma (LLC) cell membranes (CRV-LLCM). The pH/GSH-responsiveness greatly enhanced the release of dBET6 from the nanoparticles within the cells, leading to the degradation of BRD4 in both *in vitro* and *in vivo* settings.^201^ ARV-771 (BRD4 degrader) is encapsulated by a glutathione responsive polymer, leading to a nanoengineered BRD4 degrader. This enhances BRD4 degradation and reduces the expression of the downstream oncogene c-Myc. It has demonstrated superior antitumor efficacy and biocompatibility in an animal model.^202^

A nanoengineering-based biomimetic nanodrug delivery system has been designed for the targeted delivery of a KRAS–PDEδ degrader (PIPD) to pancreatic cancer cells. This approach aims to overcome the limitations of PROTAC and enhance the efficacy of PDEδ degradation. As a result, cellular apoptosis is induced (over 50% for both PC cells) and cell proliferation is suppressed through the inhibition of RAS signaling in an *in vitro* system.^203^

Nanocomposite hydrogels that incorporate PROTACs have been developed for immunotherapy applications. To enhance the immunotherapeutic effectiveness against head and neck squamous cell carcinoma (HNSCC), an injectable nanocomposite hydrogel was created. This hydrogel has a polymer framework (PLGA–PEG-PLGA) and is loaded with imiquimod-encapsulated nanoparticles and mesoporous silica nanoparticles coated with a cancer cell membrane. These nanoparticles contain a peptide-based PROTAC for BMI1 (polycomb ring finger oncogene) and paclitaxel. The PROTAC peptide is used for the degradation of BMI1 and releases paclitaxel from the pores of the particles to induce apoptosis and enhance immunotherapy. These technologies work by inhibiting both tumor growth and HNSCC metastasis through the simultaneous modulation of an immunosuppressive tumor microenvironment and degradation of BMI1.^204^

**Figure 10:**
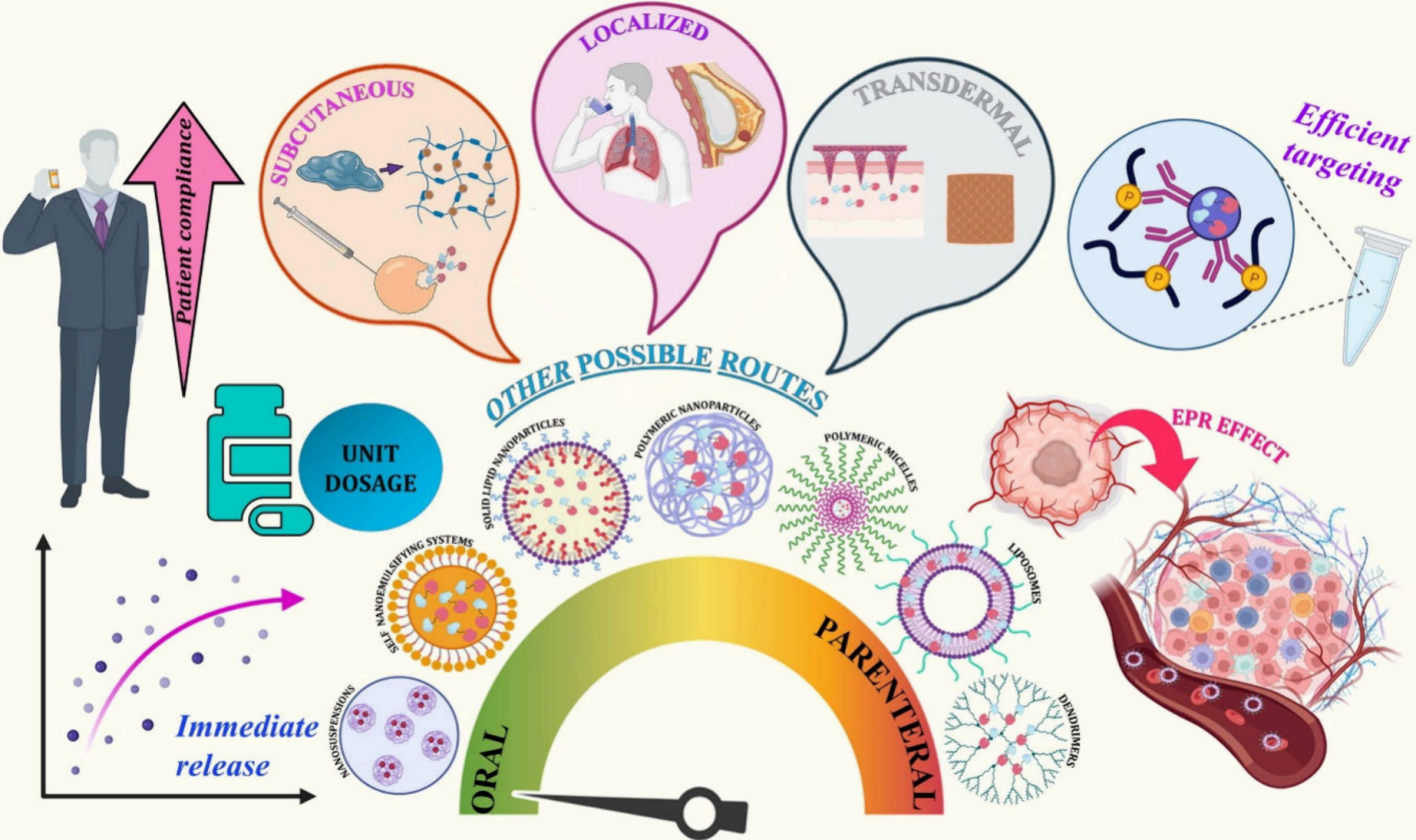
A diagram illustrating different formulation strategies to tackle the physicochemical challenges related to PROTACs is shown below. Adapted with permission from^193^ under CCBY 4.0 License.

The utilization of nanomaterials for PROTAC delivery offers a promising avenue to overcome limitations associated with bioavailability and targeted delivery. As research progresses, further exploration of biocompatible and biodegradable nanocarriers, optimization of tumor targeting strategies, and a deeper understanding of the in vivo behavior of these nano-PROTACs will be crucial for their successful clinical translation. Figure 10 illustrates the different strategies to tackle the challenges of PROTAC.

### 6.5 Tissue Specificity of PROTAC

PROTACs hold promise for achieving targeted protein degradation within specific cell types, tissues, or disease states by leveraging E3 ligases with restricted expression patterns. This approach could significantly improve the therapeutic index of PROTACs targeting essential cellular proteins. While most known PROTAC-compatible E3 ligases exhibit widespread expression, a few, like MDM2 and RNF4, show tissue specificity.^34^ Prioritizing ligand development for these tissue-restricted ligases offers a powerful strategy to minimize on-target and off-target toxicities.^34^

For example, BRD4 PROTACs targeting cancer or immune cells could avoid gut toxicity associated with pan cellular BRD4 depletion.^205^ Tissue-selective PROTACs also open doors to targeting previously intractable proteins with broad expression but severe systemic depletion effects. The success story of BCL-xL PROTACs (DT2216) exemplifies this concept. DT2216 exploits VHL, a minimally expressed ligase in platelets, to degrade BCL-xL in cancer cells, thereby avoiding the platelet toxicity observed with traditional BCL-xL inhibitors. However, a critical knowledge gap exists–how to quantify E3 ligase activity across diverse tissues and cell types. While transcriptomic and proteomic approaches provide valuable insights. Developing methods to assess E3 ligase activity will be crucial for unlocking the full potential of cell and tissue specific PROTAC development.^34^

### 6.6 Toxicities or Side Effects of PROTAC

Despite their therapeutic promise, PROTACs present unique safety challenges. Prolonged target protein degradation can lead to unintended side effects. Optimizing PROTAC design through linker and ligand modifications can control degradation kinetics and minimize this risk. Reversible PROTACs offer even greater control by allowing degradation to be halted through the addition or removal of a small molecule.

Off-target effects are another concern, as PROTACs can degrade unintended proteins, disrupting cellular pathways and causing dysfunction or immune responses.^206^ A critical difference between PROTACs and traditional inhibitors is the complete degradation of target proteins. This can lead to slow recovery times for cells, potentially causing long-lasting side effects even after drug withdrawal. Careful selection of the target protein is crucial, as exemplified by the lethal effects of complete BRD2/BRD4 degradation compared to the tolerability of BET bromodomain inhibition.^195^ Similarly, PROTACs targeting E3 ligases with essential functions in healthy cells (e.g., CRBN or VHL) can have severe consequences. The failure of CDK9 and AURKA PROTACs due to cytotoxicity highlights this point.^195^

Furthermore, competition with natural substrates for E3 ligase binding and the “hook effect” can further complicate matters. The hook effect occurs when PROTACs, at high concentrations, saturate binding sites on both the target protein and the E3 ligase. This leads to the formation of inactive binary complexes (PROTAC-target or PROTAC-E3 ligase) instead of the productive ternary complex (E3 ligase-PROTAC-target) required for target ubiquitination and degradation. This effectively hinders PROTAC activity in a concentration-dependent manner, limiting its therapeutic potential.^207^

Therefore, achieving precise control over degradation and targeting PROTACs to diseased cells is paramount for their safe clinical application. Researchers are actively developing multifunctional PROTACs with enhanced safety profiles.^208^

## 7 Conclusion

The utilization of targeted protein degradation through PROTACs characterizes a groundbreaking modernization in the field of immunotherapy. Since their inception two decades ago, PROTACs have emerged as a promising strategy for drug development, especially for oncology. This innovative approach allows for the precise modulation of immunotherapeutic strategies by selectively eliminating oncogenic proteins, inducing immunogenic cell death, disrupting immune checkpoints, and eliminating immunosuppressive cytokines. PROTAC-based immunotherapy can potentially transform the immunosuppressive tumor microenvironment into an immunologically active state. The recent advancements in PROTAC technology underscore its feasibility and efficacy in modulating key immune checkpoints and signaling pathways, thereby enhancing the body’s immune response against cancer.

Specifically, PROTACs can degrade proteins that suppress the immune system, such as PD-1 and PD-L1. By doing so, PROTACs can help to activate the immune system’s ability to fight cancer cells. PROTAC can also be used to target proteins that are involved in tumor growth, such as the androgen receptor and the EGFR. By degrading these proteins, PROTACs can help to shrink tumors and make them more susceptible to immune attack. PROTAC technology can potentially enhance the efficacy and safety of cancer immunotherapy by degrading key proteins involved in tumor immune evasion and inflammation. By combining PROTAC with other immunomodulatory agents, such as checkpoint inhibitors, cytokines, and CAR-T cells, synergistic effects can be achieved to overcome tumor resistance and improve clinical outcomes.

## 8 Future Perspectives

The future design of PROTACs should optimize their physicochemical characteristics, solubility, cellular uptake, ADME pharmacokinetics, therapeutic potency, tissue specificity, and safety profile to achieve better performance and outcomes in targeted protein degradation along with minimizing potential toxicities and side effects. The goal of PROTACs is to achieve oral bioavailability, which would greatly facilitate their clinical translation from concept to therapy. The optimization of PROTACs as effective drugs and elucidate their therapeutic mechanisms are key research priorities. PROTAC can also be applied to modulate the immune system in other diseases, such as autoimmune disorders, infectious diseases, and neurodegenerative diseases, by targeting specific proteins that regulate immune responses. PROTAC offers a novel strategy to design small-molecule immunotherapeutic that can overcome the limitations of macromolecular drugs, such as poor oral bioavailability, tissue penetration, and transmembrane transport. PROTAC can benefit from the development of new E3 ligases, linkers, and delivery systems, as well as the integration of novel technologies, such as photochemical control, molecular glues, allosteric^209^ and in-cell click chemistry,^210^ to improve the efficiency and selectivity of protein degradation. Although the development of PROTAC-based immunotherapy is still in its early stages, it has the potential to revolutionize cancer treatment. While PROTACs have shown potential in preclinical studies, their application in cancer immunotherapy is an evolving area with several perspectives for the future, such as 1) enhancing immunotherapy efficacy, 2) combination therapies, 3) personalized medicine, 4) reducing resistance, 5) minimizing off-target effects, 6) expanding targetable proteins, 7) more clinical trials and regulatory approval, and 8) technological advancements in cancer therapy. Innovative strategies such as nanoformulations and tissue-specific drug delivery systems may offer solutions to these challenges, thereby, paving the way for the clinical translation of PROTAC-based therapies. This pioneering strategy holds the potential to transform cancer treatment and enhance the quality of life for countless cancer patients. As research in this field continues to advance, it is imperative to foster interdisciplinary collaborations and investments in innovative technologies to unlock the full potential of PROTACs and pave the way for next-generation precision medicines.
